# NleG Type 3 Effectors from Enterohaemorrhagic *Escherichia coli* Are U-Box E3 Ubiquitin Ligases

**DOI:** 10.1371/journal.ppat.1000960

**Published:** 2010-06-24

**Authors:** Bin Wu, Tatiana Skarina, Adelinda Yee, Marie-Claude Jobin, Rosa DiLeo, Anthony Semesi, Christophe Fares, Alexander Lemak, Brian K. Coombes, Cheryl H. Arrowsmith, Alexander U. Singer, Alexei Savchenko

**Affiliations:** 1 Division of Cancer Genomics and Proteomics, Ontario Cancer Institute, Toronto, Ontario, Canada; 2 Department of Medical Biophysics, University of Toronto, Toronto, Ontario, Canada; 3 Banting and Best Department for Medical Research, University of Toronto, C.H. Best Institute, Toronto, Ontario, Canada; 4 Department of Biochemistry and Biomedical Sciences, McMaster University, Hamilton, Ontario, Canada; The Rockefeller University, United States of America

## Abstract

NleG homologues constitute the largest family of type 3 effectors delivered by pathogenic *E. coli*, with fourteen members in the enterohaemorrhagic (EHEC) O157:H7 strain alone. Identified recently as part of the non-LEE-encoded (Nle) effector set, this family remained uncharacterised and shared no sequence homology to other proteins including those of known function. The C-terminal domain of NleG2-3 (residues 90 to 191) is the most conserved region in NleG proteins and was solved by NMR. Structural analysis of this structure revealed the presence of a RING finger/U-box motif. Functional assays demonstrated that NleG2-3 as well as NleG5-1, NleG6-2 and NleG9′ family members exhibited a strong autoubiquitination activity *in vitro*; a characteristic usually expressed by eukaryotic ubiquitin E3 ligases. When screened for activity against a panel of 30 human E2 enzymes, the NleG2-3 and NleG5-1 homologues showed an identical profile with only UBE2E2, UBE2E3 and UBE2D2 enzymes supporting NleG activity. Fluorescence polarization analysis yielded a binding affinity constant of 56±2 µM for the UBE2D2/NleG5-1 interaction, a value comparable with previous studies on E2/E3 affinities. The UBE2D2 interaction interface on NleG2-3 defined by NMR chemical shift perturbation and mutagenesis was shown to be generally similar to that characterised for human RING finger ubiquitin ligases. The alanine substitutions of UBE2D2 residues Arg5 and Lys63, critical for activation of eukaryotic E3 ligases, also significantly decreased both NleG binding and autoubiquitination activity. These results demonstrate that bacteria-encoded NleG effectors are E3 ubiquitin ligases analogous to RING finger and U-box enzymes in eukaryotes.

## Author Summary

Many bacterial pathogens utilize a multiprotein ‘‘injection needle’’ termed the type III secretion system to deliver a set of proteins called effectors into the host cell. These effectors then manipulate host signalling pathways to the advantage of the pathogen, often mimicking eukaryote-specific activities. We present a study of an uncharacterised family of effectors called NleG, secreted primarily by enterohaemorrhagic *E. coli* (EHEC) O157:H7, a causative agent of human gastroenteritis. We solved the solution structure of a conserved C-terminal region of an NleG family member by NMR. Structural analysis demonstrated that the NleG structure is similar to the RING finger/U-box domain found primarily in eukaryotic ubiquitin ligases. The activity of these domains in eukaryotes is an essential part of the ubiquitination signalling system. Due to its central role in cell metabolism and the host immune response, the ubiquitination system has emerged as a primary target for bacterial effectors. Our biochemical analysis demonstrated that NleG proteins selectively interact with human E2 ubiquitin conjugating enzymes and exhibit *in vitro* activity typical of eukaryotic E3 ligases. Our data reveal that NleG effectors structurally and functionally mimic host U-box/RING E3 ubiquitin ligases. Future research will focus on determining targets of NleG ubiquitin ligase activity and the role in *E. coli* pathogenesis.

## Introduction

One of the best-studied molecular machineries in bacteria-host cell interactions is the Type 3 Secretion System (T3SS). The T3SS is a syringe-like multi-protein complex spanning both the inner and outer bacterial membranes and the host cell membrane. It allows various Gram-negative bacteria, including important human intra- and extracellular pathogens such as *Yersinia* sp., *Shigella*, *Salmonella* and pathogenic strains of *Escherichia coli* as well as plant pathogens such as *Pseudomonas syringae*, to inject a set of specific proteins, known as effectors, directly into the cytoplasm of the host cell. These effectors then act to alter host responses and promote the dissemination of bacteria.

There are over a hundred families of confirmed and potential effectors secreted by the T3SS [1], [2], [3]. The specific functions of most of these proteins in the host cell remain unknown. The few examples of functionally characterised effector proteins highlight their ability to target a range of key mechanisms in host cells by mimicking the eukaryote-specific activities that are often not found in prokaryote biology, such as actin polymerisation [4], [5], vesicle trafficking [6], [7] and specific signal transduction pathways unique to eukaryotes [8], [9].

The host ubiquitin proteasome system (UPS) is emerging as one of the main targets for effector proteins [10], [11], [12]. The ubiquitination system involves the specific tagging of proteins by covalent attachment of single or multiple ubiquitin polypeptide chains. This modification labels the protein either for degradation by the 26S proteasome or for other functions such as localisation to specific cell compartments.

Ubiquitination is a multi-step process that involves at least three classes of enzymes, designated E1, E2 and E3. The ubiquitin activating enzyme (E1) first charges ubiquitin in an ATP-dependent manner to form an E1-ubiquitin thioester intermediate. This activated ubiquitin is then transferred to the active cysteine of an ubiquitin conjugating enzyme (E2). The transfer of ubiquitin from the E2 enzyme to the target protein is mediated by ubiquitin-protein ligases (E3), which are responsible for the selectivity of the ubiquitination process. There are possibly hundreds of E3 ligases in a given eukaryotic cell and most of the characterised eukaryotic E3 ligases can be divided into two main classes depending of their functional domains. The first class of E3 ligases contain a HECT (homologous to E6-associated protein C-terminus) domain; these enzymes play a direct role in catalysis during ubiquitination [13]. They accept ubiquitin from E2 enzymes via the thioester bound to their active site cysteine, and then directly transfer the ubiquitin to the targeted substrates [14], [15]. The second class of E3 ligases contain a RING (really interesting new gene) or a U-box domain (a modified RING motif which lacks the Zn^2+^ ions present in RING), and act as adaptor-like molecules. This type of E3 ligase binds to an E2-ubiquitin complex and to the target protein and activates the transfer of the ubiquitin cargo from the former to the latter [16], [17].

While bacteria lack the UPS, bacterial pathogens secrete many effectors with UPS-specific functions [10], [11], [12]. The largest and most diverse group of effectors known to associate with the host UPS are those able to mimic the E3 ubiquitin ligase activity. This category includes the *P. syringae* pv. *tomato* (strain DC3000) T3SS effector AvrPtoB, which provokes the hypersensitive response in tomato plants expressing the Pto resistance gene [18]. Despite the fact that AvrPtoB does not share any significant sequence similarity with eukaryotic E3 ligases, its C-terminal domain is structurally similar to the U-box domain and functions as an E3 ligase [19]. The role of AvrPtoB has been linked with proteasome dependent degradation of several plant protein targets, including tomato kinase Fen [20]. The plant pathogen *Ralstonia solanacearum* encodes for seven effectors of the GALA family which have the F-box motifs that bind the host Skp1 proteins and function as SCF (Skp1, Cullin, F-Box) E3 ligases [21]. The *Salmonella* SopA effector is involved in the regulation of the host inflammatory response and demonstrated functional and structural features similar to the HECT E3 ligases [22]. Finally, members of the IpaH effector family, found in diverse plant and animal pathogens and symbionts including *Pseudomonas* sp., *Salmonella* and *Shigella*, comprise a novel class of E3 ligases [23]. The *Salmonella* representative of this family, SspH1, binds to and ubiquitinates the mammalian protein kinase PKN1 [23], [24]. The role of IpaH family proteins in pathogenesis and the identification of their eukaryotic targets have yet to be elucidated. The conserved C-terminal domains of these proteins contain an invariant cysteine that forms a Cys-Ub intermediate similar to the active site Cys of HECT E3 ligases [25], [26]. However, according to the recently published structures of three members of this family, the *Shigella* IpaH1.4 [26] and IpaH3 [25] effectors and *Salmonella* SspH2 [27] the C-terminal domain of these effectors does not share any structural similarity with HECT E3 ligases. The N-terminal regions of the IpaH family members contain diverse leucine-rich repeat (LRR) domains apparently responsible for specificity toward different substrates in the host cell and autoregulation of E3 activity [23], [24], [26], [27].

The attaching and effacing human pathogen enterohaemorrhagic *Escherichia coli* (EHEC) is the causative agent of diarrheal disease characterised by bloody diarrhoea and haemolytic uremic syndrome. In contrast to intracellular pathogens, EHEC use a set of effectors delivered by the T3SS to subvert actin polymerization to form the so-called “pedestal” on the host-cell surface which facilitates adherence and colonisation of the pathogen [4]. The T3SS apparatus of EHEC is encoded within a specific locus in its genome named the “locus of enterocyte effacement” or LEE [28]. Effector proteins identified within the LEE were thought to represent the complete EHEC effector repertoire, since the transfer of this pathogenicity island alone was sufficient to allow the non-pathogenic *E. coli* K12 to induce an attaching and effacing-like phenotype [29]. However, the recent genome-wide analysis of EHEC O157:H7 revealed a large number of additional non-LEE-encoded (Nle) effector proteins, totalling over 20 protein families [30]. The largest family among these newly identified effectors was called NleG. The Sakai strain of O157:H7 contains 14 different representatives of this family and over 20 NleG homologues can be found in different strains of pathogenic *E. coli* and *Salmonella*. NleG proteins have been demonstrated to be substrates of the T3SS and to be translocated into human cells [30], [31]. The NleG family shares no sequence homology to proteins of known function. In the current study, we demonstrate that the most conserved C-terminal portion in NleG effectors is structurally similar to the RING finger/U-box motif and that these effectors function *in vitro* as E3 ubiquitin ligases.

## Results

### Identification of the conserved C-terminal domain in NleG proteins

**Figure 1:**
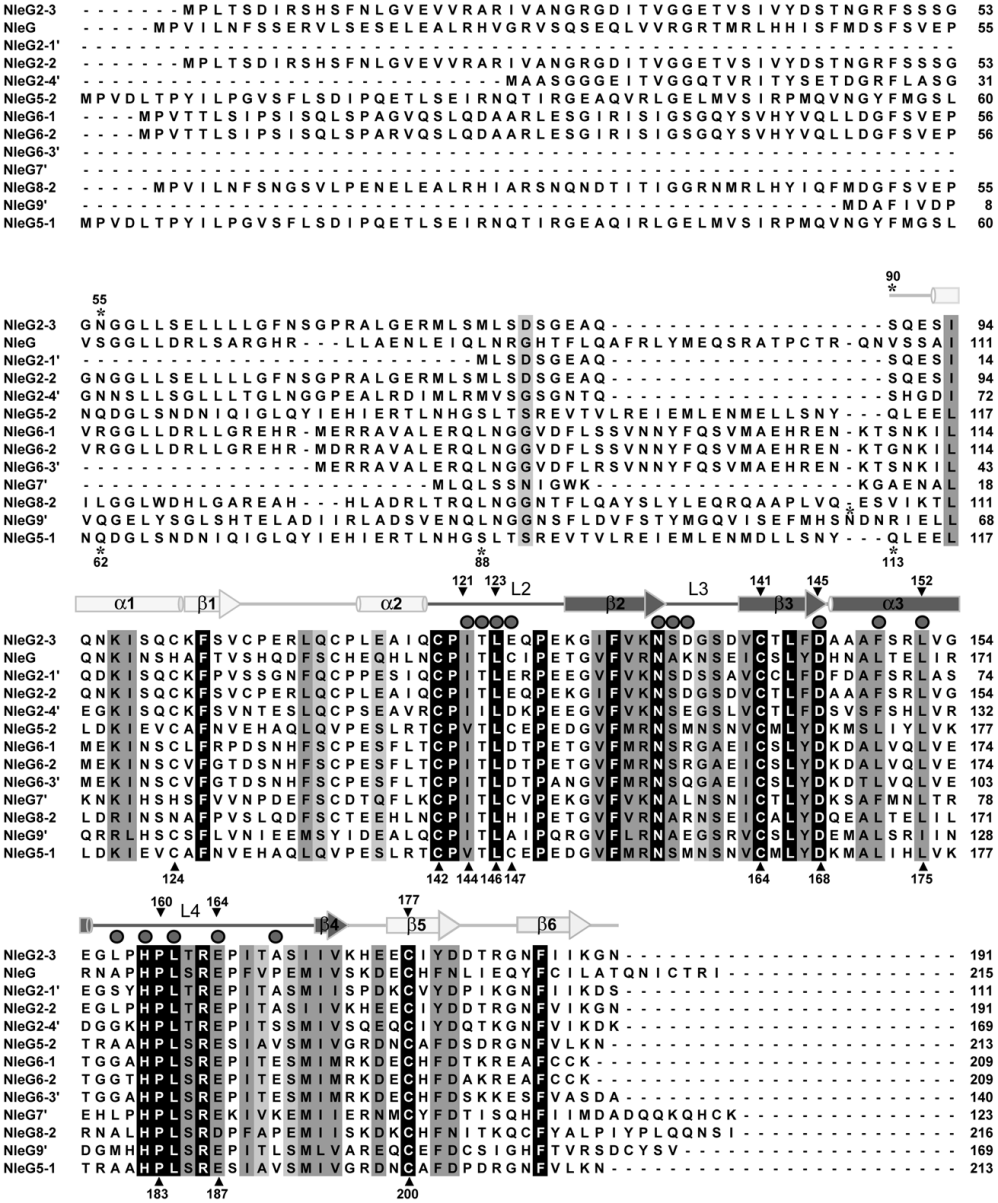
Multiple sequence alignment of NleG effectors. The amino acid sequence of NleG2-3 (Sakai ID: ECs2156), NleG (ECs1824), NleG9′ (ECs1828), NleG2-2 (Ecs1994), NleG2-4′ (ECs2229), NleG5-2 (ECs2154), NleG6-1 (ECs1995), NleG6-2 (ECs2155), NleG6-3′ (ECs3488), NleG7′ (ECs2226), NleG8-2 (ECs3486), NleG2-1′ (ECs1811), and NleG5-1 (ECs1996) were aligned using ClustalX [59]. The secondary structure of the NleG2-3[90–191] fragment, as determined by NMR, is shown above the alignment. Secondary elements corresponding to the RING/U-box motif are shaded darkly, while other secondary elements are shaded lightly. Mutation sites are designated with shaded triangle above the alignment when made in NleG2-3 and under the alignment when NleG5-1 was used as template. The positions of the N-terminal amino acid of NleG C-terminal fragments used in this study are denoted by an asterisk. Residues which were line-broadened upon addition of UBE2D2 are denoted by dark grey spheres above the alignment.

The Pfam database (http://pfam.sanger.ac.uk) identifies 24 NleG homologues found mainly in pathogenic *E. coli* and *Salmonella* genomes. This group of proteins form a distinct family, dubbed DUF1076 (PF06416), which does not share any significant sequence similarity with other proteins. NleG family members demonstrate significant variation in length (from 111 to 223 amino acids) and sequence, featuring as low as 28% sequence identity between certain family members. Nevertheless, a comparative sequence analysis demonstrated that all NleG proteins contain a conserved region of ∼100 residues localised to the C-terminus (Figure 1). The N-terminal part of NleG proteins appears to be significantly less conserved.

**Figure 2:**
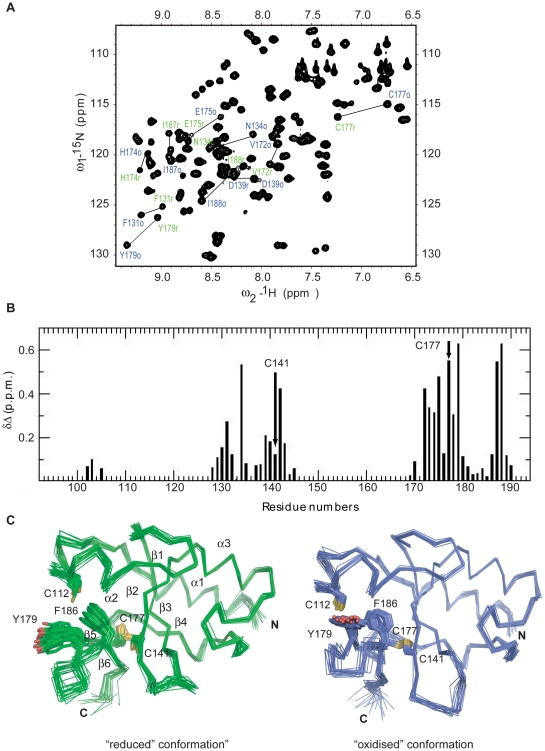
Solution structure of NleG2-3[90–191] (A) 2D ^15^N-HSQC spectrum of NleG2-3[90–191] at 25°C and pH 7.0. Selected peaks corresponding to reduced and oxidised conformations are labeled as r (green) and o (blue), respectively. (B) Chemical shift difference (Δ_ppm_) between NleG2-3[90–191] reduced and oxidised conformations plotted against the residue number. Δ_ppm_ was calculated using the formula Δ_ppm_ = [(Δδ^2^_HN_ + (Δδ_N_/5)^2^]^1/2^, where δ_N_ and δ_HN_ are the chemical shift differences for ^1^HN and ^15^N between the two forms, respectively. The positions of Cys141 and Cys177 forming the disulfide bond are denoted. (C) Ensemble of 20 refined structures of NleG2-3[90–191] in the reduced (left) and oxidised (right) conformations, with the positions of side chains of residues with different conformations between the two forms highlighted. Positions of secondary structure elements on the reduced conformation are denoted.

Based on this analysis, we postulated that the conserved C-terminal region of NleG proteins might represent a distinct independently-folding domain. To test this hypothesis, the corresponding fragment of NleG2-3 from the *E. coli* O157:H7 strain, spanning from amino acid 90 to 191 (NleG2-3[90–191]; Figure 1) was expressed in ^15^N enriched minimal medium and purified to homogeneity (see Methods for details). The NleG2-3[90–191] protein fragment showed very good signal dispersion in ^1^H/^15^N-heteronuclear single quantum correlation (HSQC) NMR spectra (Figure 2A). The number of resolved amide proton peaks was sufficient to account for all backbone resonances indicating that this protein fragment is a distinct, independently folded domain amenable to NMR structure determination.

### Solution structure of the NleG2-3 C-terminal domain

Analysis of the NleG2-3[90–191] ^1^H/^15^N-HSQC spectrum suggested that 57 residues (56% of the total) of NleG2-3[90–191] exhibited two sets of amide peaks of approximately equal intensity, indicative of an equimolar mixture of two conformations (Figure 2A). Complete assignments were obtained for all resonances that appeared in the ^1^H/^15^N-HSQC spectrum by the combined use of the automated assignment program FAWN (Lemak and Arrowsmith, unpublished) and manual analysis.

The assignment of backbone resonances identified two regions with the largest chemical shift differences between the two conformations; these span NleG2-3 residues 128 to 143 and residues 172 to 190 (Figure 2A and 2B). Each of these regions contains a conserved cysteine residue, at positions 141 and 177, respectively. The analysis of ^13^Cβ chemical shifts indicated that Cys141 and Cys177 appear in solution in equal populations of reduced and oxidised forms (31.96 ppm and 46.11 ppm for Cys141; 31.02 ppm and 45.61 ppm for Cys177, respectively), suggesting that while the data collection was performed under reduced conditions (in the presence of 10 mM DTT, see Methods for details) the source of the heterogeneity in ^15^N-HSQC spectra is the formation of a disulfide bond involving these residues. The NleG2-3[90–191] fragment contains six cysteine residues, three of which (Cys119, Cys141 and Cys177) are absolutely conserved in all NleG homologues (Figure 1). The analysis of ^13^Cβ shifts demonstrated that all cysteine residues except for Cys141 and Cys177 appear only in the reduced form (29.31 ppm for Cys101, 28.21 ppm for Cys106, 29.75 ppm for Cys112, 30.20 ppm for Cys119). Therefore only Cys141 and Cys17are involved in formation of a disulfide bond. From ^15^N spin relaxation measurements, the correlation time of the NleG2-3[90–191] fragment was estimated to be 5.9 ns (corresponding to a spherical protein of 12 kDa). This implies that this protein fragment is monomeric in solution and that the disulfide bond between Cys141 and Cys177 is intramolecular rather that intermolecular. Mutation of either Cys141 or Cys177 to alanine resulted in a reduction in the number of peaks in the ^1^H/^15^N-HSQC spectrum to a single set (Figure S1), confirming that the heterogeneity observed in the spectrum of the wild-type NleG2-3[90–191] fragment is due to the presence of the two species in the sample, one with and one without an intramolecular disulfide bond between these cysteine residues.

In order to gain a structural insight onto both “reduced” (featuring Cys141 and Cys177 in the reduced form) and “oxidised” (featuring Cys141 and Cys177 forming a disulfide bond) conformations of the NleG2-3[90–191] fragment, no further effort was made to separate these forms in the protein sample over the course of solution structure determination. Instead the NMR data were collected on the protein sample containing a mixture of these conformations, and both conformations of the NleG2-3[90–191] domain were solved independently.

The NMR solution structures of the “oxidised” and “reduced” NleG2-3[90–191] were generated using automated noeassign/CYANA iterative calculations and further refined by including residual dipolar coupling restraints and restrained molecular dynamic simulations in explicit solvent with the program CNS. The overall quality of the fit of the residual dipolar couplings (RDC) to the NleG2-3[90–191] structures after refinement, as indicated by quality factors (Q) of 0.113 (65 ^1^D_NH_), 0.206 (68 ^1^D_CaCo_) and 0.156 (53 ^1^D_NCo_) for the “reduced” conformation, and 0.102 (64 ^1^D_NH_), 0.179 (54 ^1^D_CaCo_) and 0.137 (48 ^1^D_NCo_) for the “oxidised” conformation, respectively (Figure S2), indicate that the NMR structures provide a good description of the 3D structure of the NleG2-3[90–191] domain in solution. CS-Rosetta calculations provide excellent independent cross-validation for the accuracy of the solution structures of NleG2-3[90–191]. Backbone superimposition of the best cluster of CS-Rosetta models with reduced and oxidised NleG2-3[90–191] from residues 91 to 189 gave an r.m.s deviation of 1.9 Å and 1.1 Å, respectively. Thus, solution structures and Rosetta structures are all in good agreement with respect to the global folding of the NleG2-3[90–191] fragment. The structural statistics for both conformations are summarized in Table 1.

**Table 1:** Structural statistics for the ensemble calculated for NleG2-3[90–191] solution structure.

	NleG2-3[90–191] “reduced” conformation		NleG2-3[90–191] “oxidized” conformation	
Distance restraints				
All	2806		2949	
Intra-residue (i = j)	471		492	
Sequential (|i-j| = 1)	661		657	
Medium range (2≤|i–j|≤4)	567		591	
Long range (|i–j|>4)	1041		1138	
Hydrogen bonds	33×2		34×2	
Disulfide bond	-		3	
Dihedral angle restraints				
All	164		160	
φ	82		80	
ϕ	82		80	
Residual dipolar coupling restraints				
All	186		166	
^1^D_NH_	65		64	
^1^D_NCo_	53		48	
^1^D_CaCo_	68		54	
CYANA target function, (Å^2^)	0.91±0.007		1.89±0.019	
Number of violations				
Distance restraints (>0.5 Å)	0		0	
Dihedral angle restraints (>5°)	0		0	
r.m.s.d from experimental restraints				
Distance (Å)	0.0168±0.0015		0.0172±0.0010	
Dihedral angle (°)	0.5375±0.1007		0.4074±0.1405	
r.m.s.d from idealized covalent geometry				
bond (Å)	0.0135±0.0002		0.0136±0.0001	
bond angles (°)	0.9231±0.0182		0.9099±0.0124	
CNS energy (kcal/mol)				
Total	−3100±85		−3011±76	
Van der Waals	−710±8		−721±7	
Electrostatic	−3697±83		−3596±79	
r.m.s.d from mean structure^a^				
Backbone atoms	0.45±0.07		0.36±0.06	
All heavy atoms	0.86±0.11		0.75±0.11	
Ramachandran plot (%)^a^				
Residues in most favored regions	89.6		87.5	
Residues in additional allowed regions	9.9		11.3	
Residues in generously allowed regions	0.5		0.0	
Residues in disallowed regions	0.1		1.1	
Global quality scores^b^	Raw	Z-score	Raw	Z-score
Verify3D	0.43	−0.48	0.37	−1.44
ProsaII	0.72	0.29	0.83	0.74
Procheck (phi-psi)^a^	−0.32	−0.94	−0.36	−1.10
Procheck (all)^a^	−0.23	−1.36	−0.27	−1.60
MolProbity clash	17.31	−1.44	21.41	−2.15
RPF scores^c^				
Recall	0.939		0.942	
Precision	0.957		0.942	
F-measure	0.948		0.942	
DP-score	0.831		0.820	

NleG2-3[90–191] consists of an ensemble of the 20 lowest energy structures out of 100 calculated.

a Rmsd values for residues 91–189.

b Calculated from NESG PSVS program [56].

c RPF scores were defined and calculated by using RPF program [51].

The oxidised and reduced NleG2-3[90–191] conformations had an identical overall fold and superimpose with an r.m.s. deviation of 1.4 Å. The beta carbon atoms of Cys141 and Cys177 forming a disulphide bridge in the oxidised form remain in close proximity in the reduced form (i.e. 3.8 Å), suggesting that the interchange between the two observed conformations requires very little structural alteration. The major difference between the two NleG2-3[90–191] conformations involves a movement in the oxidised structure which brings the C-terminal β-hairpin closer to the long loop (L1) between strand β1 and helix α2. Consequently, in the oxidised conformation the side chain of Tyr179 aligns with that of Phe186 filling the space between these elements, while in the reduced conformation the Tyr179 side chain is exposed to the solvent (Figure 2C).

### NleG2-3 C-terminal domain contains a RING finger/U-box motif

**Figure 3:**
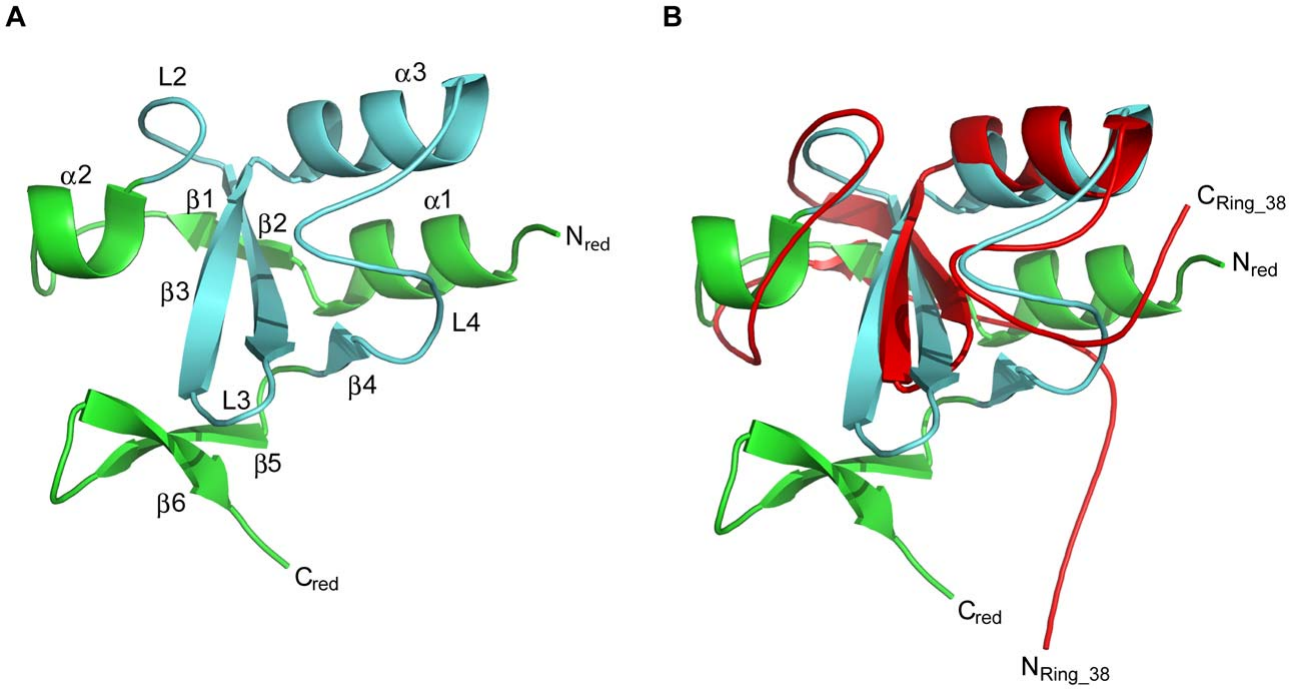
Overall ribbon diagram of NleG2-3[90–191]. Secondary elements corresponding to the RING/U-box motif are coloured cyan, while other elements of the structure are coloured green. (A) Overall structure of reduced NleG2-3[90–191] with the N- and C-terminus and secondary structure elements labelled. (B) Superimposition of reduced NleG2-3[90–191] (same as (A)) with RING finger 38 protein (PDB 1X4J, red); the N- and C-termini of both molecules labelled.

The analysis of the NleG2-3[90–191] structure revealed the presence of a motif similar to RING finger/U-box domains. This part of the NleG2-3[90–191] structure, spanning residues 101 to 172 includes a three-stranded β-sheet (β2–β3, residues 129–145, and a strand β4, abbreviated to residues 171–172), an α-helix (helix α2, residues 146–155) and the loops connecting these elements, dubbed L2, L3 and L4 (Figures 1 and 3A). The tertiary structure comparison server DALI [32] confirmed the strong similarity of this region of the NleG2-3[90–191] structure with several structures of RING-finger/U-box domains, including that of Pre-mRNA splicing factor Prp19 (PDB 2BAY, Z-score 5.2), RING finger 38 protein (PDB 1X4J, Z-score 5.0) and the *P. syringae* effector AvrPtoB C-terminal domain (PDB 2FD4, Z-score 4.6). The NleG2-3[90–191] structure in the reduced form superimposes onto the RING finger 38 with an r.m.s. deviation of 2.3 Å overall except for the very C-terminus of NleG2-3[90–191], corresponding to the β-hairpin (β5 and β6 strands), this extreme C-terminus does not have counterparts in otherwise homologous RING finger structures (Figure 3B).

As mentioned previously, the RING finger is a zinc-chelating domain found in E3 ubiquitin ligases. The zinc atoms in RING domains are bound through a set of conserved cysteine/histidine residues in loops L2 and L4, and are essential for correct folding and biological activity of the RING domain [16]. In the case of the U-box, zinc ions are absent so the equivalent fold is maintained by non-covalent interactions [17]. Analysis of the NleG2-3[90–191] structure did not reveal any potential metal binding sites analogous to those found in RING finger motifs. The superimposition of NleG2-3[90–191] and RING finger structures demonstrated that the conserved cysteine and histidine residues in the NleG2-3[90–191] domain mostly do not correspond to the metal binding residues in RING domains (see Figure S3A). Finally the expression of an NleG2-3[90–191] fragment in minimal medium lacking Zn^2+^ ions did not affect its ^1^H/^15^N-HSQC spectrum and the titration of Zn^2+^ ions into this protein sample did not produce any significant changes in chemical shifts (Figure S3B) features that have been observed for metal dependent RING domains [33]. This analysis suggests that the NleG2-3[90–191] fold, like those of the eukaryotic U-box domains and the AvrPtoB C-terminus, does not depend on the presence of metal ions.

Apart from the “core” RING-finger/U-box motif described above, the NleG2-3[90–191] structure contains an N-terminal α-helix (helix α1) which packs against helix α3 and a C-terminal β-hairpin (strands β5 and β6, residues 177–188) (Figure 3A and 3B). These elements do not have counterparts in the otherwise similar RING-finger/U-box structures mentioned above. Notably the intra-molecular disulfide bond formed between residues Cys141 and Cys177 in the oxidised NleG2-3[90–191] conformation connects the C-terminal β-hairpin to the central β-sheet, and may contribute to the stability of its RING finger motif. However, in the reduced conformation, the local positions of the cysteine backbone and Cβ atoms remain very similar to that in the oxidised form (the Cβ's of both Cys141 and Cys177 in the reduced and oxidised forms superimpose to 1.6 Å). Helix α1 in the NleG2-3[90–191] structure may also contribute to stabilizing the “core” RING-finger/U-box motif by reducing the solvent accessibility of its central β-sheet.

To conclude, the solution structure of the NleG2-3[90–191] fragment revealed the presence of a RING-finger/U-box motif. This motif corresponds to the most conserved portion of NleG homologues suggesting that this structural element is common among the members of this family.

### NleG proteins demonstrate autoubiquitination activity *in vitro*

**Figure 4:**
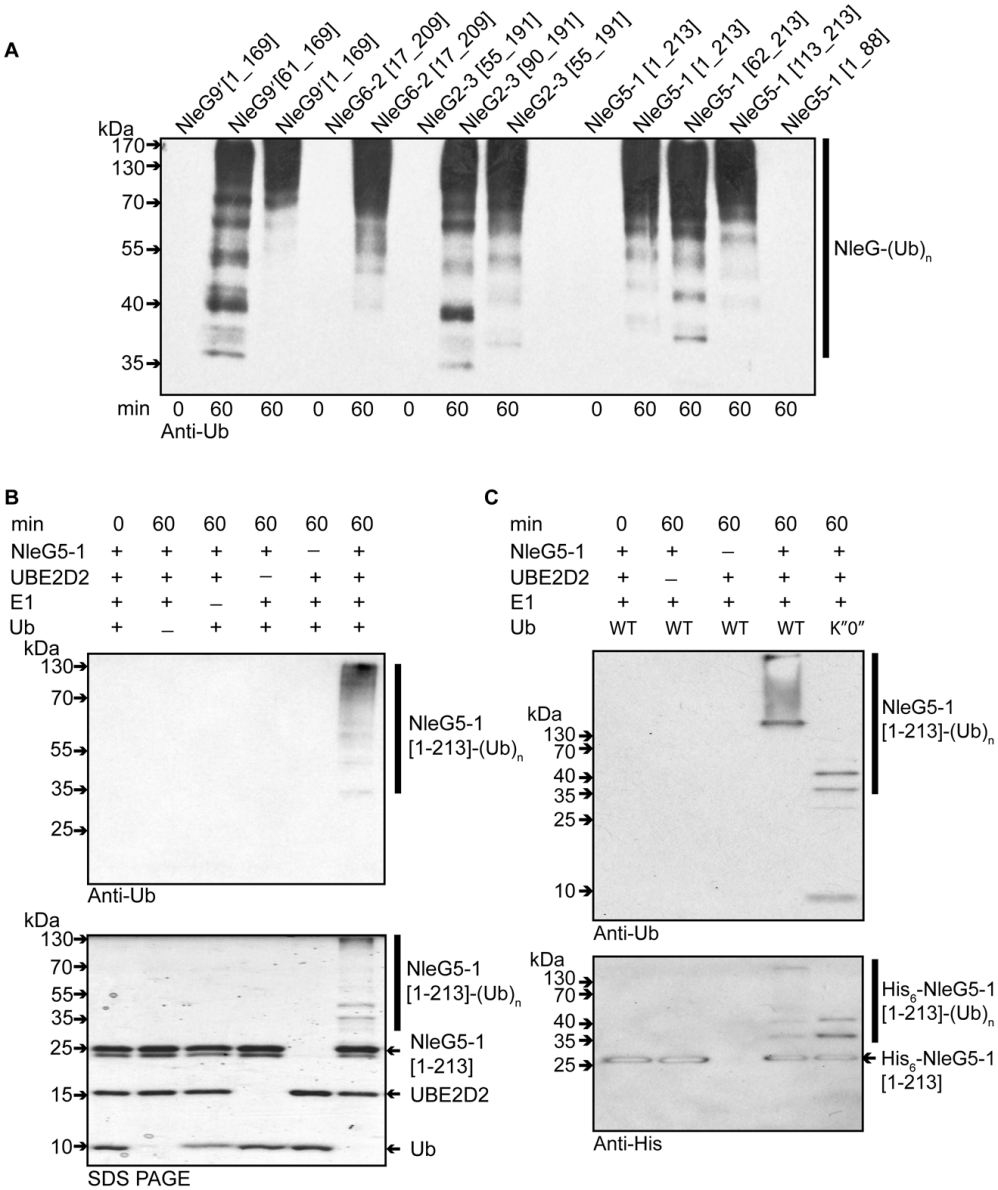
*In vitro* activity of NleG proteins. (A) Immunoblot analysis using anti-ubiquitin antibodies of reactions performed in the presence of ATP, ubiquitin, E1, UBE2D2 and full length or C-terminal fragments of His_6_-NleG isoforms. (B) Immunoblot analysis with anti-ubiquitin antibodies (top panel) and SDS PAGE electrophoresis (bottom panel) of reactions performed with ATP and in the presence or absence of ubiquitin, E1, UBE2D2 E2 and NleG5-1[1–203]. (C) Immunoblot analysis with anti-ubiquitin antibodies (top panel) and anti-His_6_ antibodies (bottom panel) of reactions performed with E1, wild type ubiquitin (WT) or ubiquitin derivative (K”O”) in the presence or absence of His_6_-NleG5-1[1–203] and UBE2D2 E2 enzyme. All reactions were incubated at 30°C for the times indicated.

The RING-finger/U-box domains in eukaryotic E3 ubiquitin ligases are primarily involved in recruiting and allosteric activation of the ubiquitin-charged E2 enzyme [16]. As a consequence of this function, RING-finger/U-box domains are able to promote autoubiquitination *in vitro* in the presence of E1, E2, ubiquitin and ATP; an assay which is commonly used to show E3 ubiquitin ligase activity, particularly when the target of the E3 ligase is not known [34]. To investigate if NleG effectors possess such an activity, the full length NleG5-1[1–213], and NleG9′[1–169] proteins, NleG fragments lacking the predicted N-terminal secretion motif (NleG5-1[62–213], NleG6-2[17–209], NleG9′[61–169] and NleG2-3[55-191]) and NleG fragments corresponding to the conserved C-terminal region (NleG5-1[113–213] and NleG2-3[90–191]) (Figure 1) were all tested in ubiquitination assays *in vitro*. As a negative control we also tested an N-terminal fragment lacking the conserved C-terminal domain (NleG5-1[1–88]). All ubiquitination reactions contained human E1, UBE2D2 E2 enzyme, which is one of the most versatile enzymes of this class, ubiquitin and ATP. Western blot analysis using anti-ubiquitin antibodies allowed visualization of a characteristic pattern corresponding to multiple polyubiquitinated protein species in the case of full-length NleG proteins as well as all C-terminal fragments (Figure 4A). In contrast no ubiquinated protein species were detected with the NleG5-1[1–88] fragment. The accumulation of polyubiquitinated species was dependant on the presence of each component of the reaction including E1 and E2 enzymes (Figure 4B), suggesting a general mechanism similar to that established for eukaryotic E3 RING finger/U-box ubiquitin ligases. Western blot analysis of products of the His_6_-NleG5-1[1–213] which represented the only polyhistidine tagged protein in the ubiquitination reaction, using anti-polyhistidine antibodies indicated that ubiquitinated protein species corresponded in part to mono- and poly- ubiquitinated His_6_-NleG5-1[1–213] protein (Figure 4C). On the other hand significant amount of high molecular weight reaction products detected by anti-ubiquitin antibodies were not recognised by anti-polyhistidine antibodies. Mass spectrometry analysis (data not shown) of His_6_-NleG5-1[1–213] ubiquitination reaction products identified the presence of Lys48 and Lys63-linked polyubiquitin chains indicating that the high molecular weight products detected by anti-ubiquitin but not by anti-polyhistidine antibodies correspond primarily to unanchored polyubiquitin chains. This finding was also supported by the analysis of products of His_6_-NleG5-1[1–213] ubiquitination reaction, where the ubiquitin K″O″ variant was used instead of wild type ubiquitin (Figure 4C). The use of K″O″ variant, in which all lysine residues are mutated, prevented the formation of polyubiquitin chains resulting in complete absence of high molecular weight protein species detected by anti-ubiquitin antibodies. The only ubiquitinated products detected in reaction using K″O″ ubiquitin expectably corresponded to the mono- and multisite ubiquitinated His_6_-NleG5-1[1–213] protein species. The formation of unanchored polyubiquitin chains is often observed as a by-product of *in vitro* ubiquitination assays of eukaryotic E3 ubiquitin ligases in the absence of specific ubiquitination substrate suggesting that autoubiquitination may not be part of physiological function of NleG5-1.

**Figure 5:**
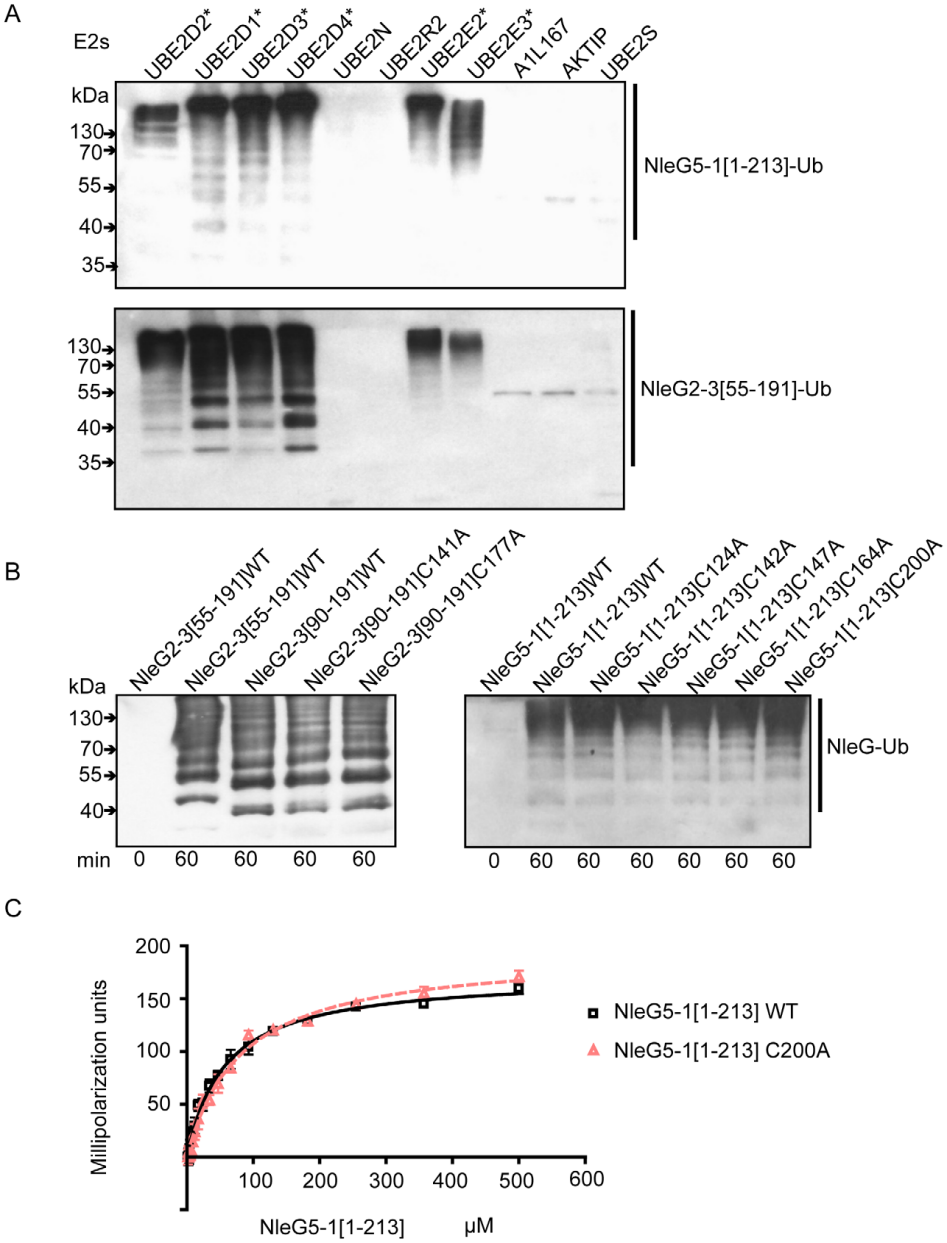
Interaction of NleG2-3 and NleG5-1 with human E2 enzymes. (A) Western blot analysis of reactions using anti-ubiquitin antibodies performed in presence of ATP, ubiquitin, E1, the indicated human E2 enzymes and either NleG5-1[1–213] (top panel) or NleG2-3[55–191] (bottom panel). Reactions were performed at 30°C for 2 hours. The asterisk indicates the E2 enzymes supporting formation of multiple ubiquitinated protein species. The E2 nomenclature is in accordance with that used by the Human Genome Organization (http://www.genenames.org/genefamily/ube2.php). (B) Immunoblot analysis using anti-ubiquitin antibodies of reactions performed in the presence of ATP, ubiquitin, E1, UBE2D2 and NleG2-3 (left) or NleG5-1 (right) variants. The NleG2-3 variants include the wild type (WT) fragments NleG2-3[55–191] and NleG2-3[90–191] and C141A and C177A mutants of NleG2-3[90–191]. The NleG5-1 variants included wild type NleG5-1[1–213] and its C124A, C142A, C147A, C164A and C200A mutants. The samples were incubated at 30°C for the times indicated at the bottom of the panel. (**C**) Determination of dissociation constants of UBE2D2 with NleG5-1[1–213] wild type (WT) or its C200A variant. The change in fluorescence polarisation of fluorescein-labeled UBE2D2 is plotted as a function of NleG5-1 concentration with error bars indicating one standard deviation.

There are at least thirty E2 enzymes encoded in the human genome and each E3 ubiquitin ligase is usually selective for only a few specific E2 enzymes. To investigate the selectivity of NleG effectors, we tested 30 purified human E2s (for a full list of tested human E2 enzymes see Table S1) for their ability to support autoubiquitination of NleG2-3[55–191] and NleG5-1[1–213], which share only 32% sequence identity. Results from Western blotting with anti-ubiquitin antibodies, demonstrated that these NleG proteins have identical E2 specificity profiles (Figure 5A). In addition to UBE2D2, UBE2D1, UBE2D3, UBE2D4 (which are all highly homologous), UBE2E2 and UBE2E3 were also able to support polyubiquitination of both selected NleG proteins. These data suggest that members of the NleG effector family may share similar selectivity for human E2 enzymes.

We investigated if the ability to form an intramolecular disulfide bond in NleG2-3[90–191] is critical for its autoubiquitination activity *in vitro*. NleG2-3[90–191] C141A and C177A mutants were compared with the wild type NleG2-3[90–191] in an autoubiquitination reaction also containing human E1 and UBE2D2 E2 enzyme. There were no significant differences in activity of the two cysteine mutants compared to the wild type (Figure 5B). We also mutated each of the five cysteine residues (Cys124, Cys142, Cys147, Cys164 and Cys200) in the context of the full-length NleG5-1 paralogue, which was more easily purified compared with full-length NleG2-3; Cys164 and Cys200 correspond to Cys141 and Cys177 in NleG2-3 (Figure 1). All five NleG5-1[1–213] mutants supported formation of polyubiquitinated protein species at a rate comparable to the wild type (Figure 5C), suggesting that none of the cysteines plays an obligatory catalytic role. These results combined with the fact that most of the eukaryotic cellular compartments, the “mise en scène” for NleG proteins, are reducing environments indicating that the reduced conformation of NleG2-3 may be prevalent *in vivo*.

In conclusion, the functional assays demonstrated that at least four NleG effectors display strong autoubiquitination activity *in vitro* characteristic to E3 ubiquitin ligases. This activity was localised to their conserved C-terminal portion, which according to the NleG2-3[90–191] structure, features a RING/U-box motif.

### Interaction of NleG5-1 and UBE2D2

Human UBE2D2 E2 enzyme can support the *in vitro* activity of all four of the NleG paralogues tested, namely NleG2-3, NleG5-1, NleG6-2 and NleG9. To characterise the molecular details of the interaction of the NleG family and UBE2D2, the UBE2D2 protein was labelled with fluorescein-5-maleimide and the change in fluorescence polarization was monitored upon titration with wild type NleG5-1[1–213] and the corresponding C200A mutant (Figure 5C). The K_d_ values for wild-type NleG5-1[1–213] and the C200A mutant were 56±2 and 81±3 µM, respectively, suggesting that the disulfide bond formation in full length NleG5-1 is not critical for its interaction with UBE2D2. These results are consistent with the fact that the reduced and oxidised conformations of the NleG2-3[90–191] have very similar structures and that they are both functional in ubiquitination assays.

**Figure 6:**
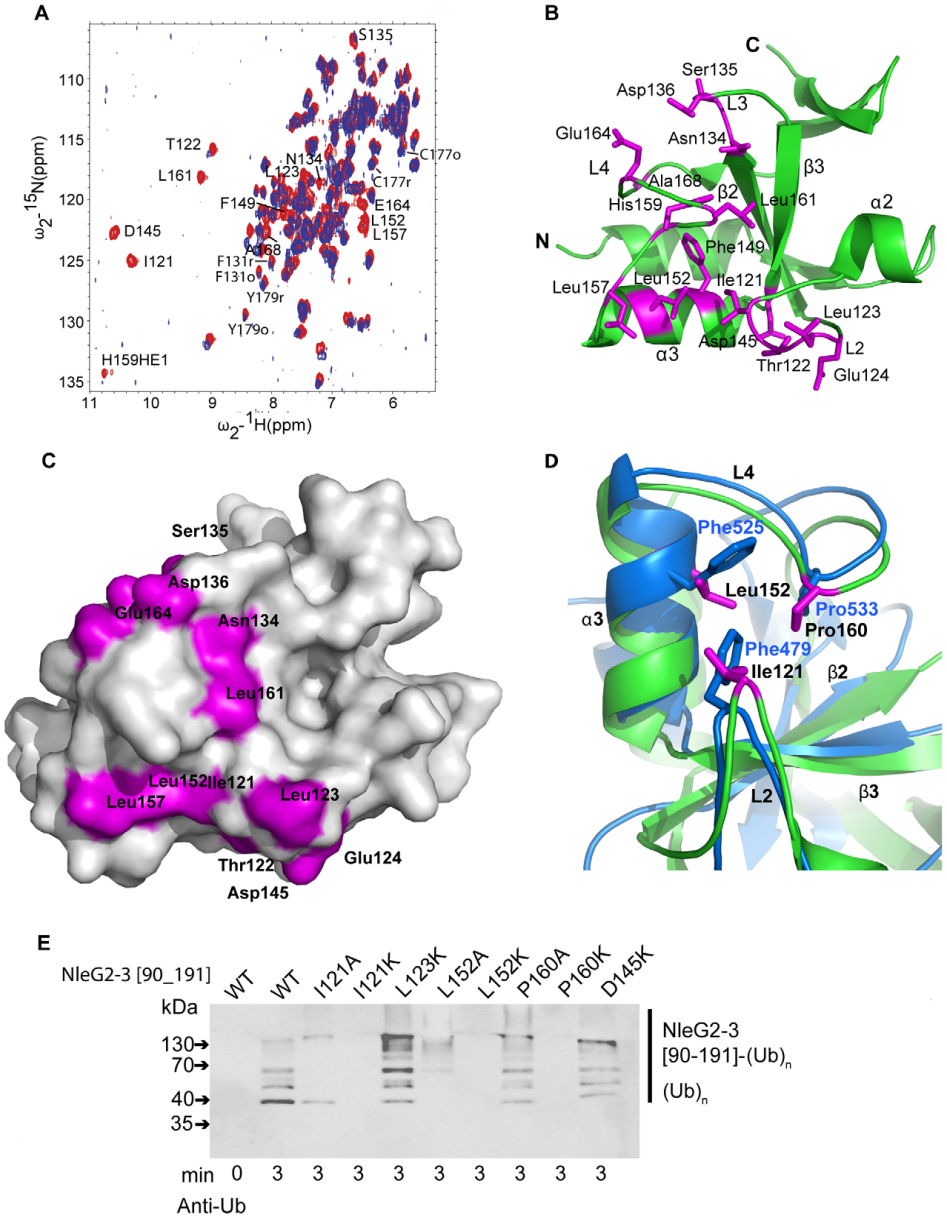
Detailed analysis of NleG interactions with UBE2D2. (A) Overlay of the ^1^N-^1^H HSQC spectrum of ^15^N-labeled NleG2-3[90–191] recorded at 25°C in the absence (red) and in the presence (lilac) of unlabeled UBE2D2 at a 1:1 protein ratio. The resonances of NleG2-3 residues significantly affected by UBE2D2 are labeled. (B) Ribbon diagram of the structure of reduced NleG2-3[90–191] (green) with residues whose NH resonances were severely line-broadened in ^1^N-^1^H HSQC spectra upon addition of UBE2D2 coloured purple. (C) Surface representation of (B). (D). Superimposition of AvrPtoB (PDB 2FD4, contact residues and backbone in bleu) and reduced NleG2-3[90–191] (predicted contact residues, purple and backbone, green), with both structures shown by ribbon diagrams, with selected secondary structure elements of NleG2-3[90–191] and selected residues from both structures labelled. (E) Immunoblot analysis using anti-ubiquitin antibodies of reactions performed in the presence of ATP, ubiquitin, E1, UBE2D2 and NleG2-3[90–191] variants. The NleG2-3 variants include the wild type (WT) fragments NleG2-3[55–191] and I121A, I121IK, L123K, L152A, L152K, P160A, P160K, E164K and D145K mutants.

To map the interactions of UBE2D2 with NleG effectors, we carried out an NMR chemical shift perturbation experiment in which unlabeled UBE2D2 protein was titrated into a sample of ^15^N-labeled NleG2-3[90–191]. The analysis of ^15^N-^1^H correlations in the resulting HSQC spectra demonstrated that upon titration of UBE2D2, the peaks corresponding to NleG2-3 residues Ile121 to Glu124, Asn134 to Asp136, Asp145, Phe149, Leu152, Leu157, His159, Leu161, Glu164 and Ala168 disappeared due to strong line broadening, indicating that these residues may be affected by interactions with the UBE2D2 enzyme (Figure 6A and 6B).

As described above the ^15^N-labeled NleG2-3[90–191] sample represents an equilibrium of the structurally similar reduced and oxidised conformations. We examined if these conformations demonstrate any detectable differences in their interactions with UBE2D2. Such differences would manifest in dissimilar rate of broadening between oxidised and reduced ^15^N-^1^H correlations of NleG2-3[90–191] residues upon titration of UBE2D2 protein. We measured both the peak height and volume of ^15^N-^1^H correlations of Phe131, Cys177 and Tyr179 NleG2-3 residues, which are the three resonances showing minimal overlap with other resonances in both their reduced and oxidised forms over the course of the titration (Figure 6A). Within the error of these measurements, line broadening of their oxidised and reduced resonances upon titration of UBE2D2 occurs at the same rate (data not shown). Thus no significant difference in interactions with UBE2D2 enzyme was detected for the reduced or oxidised conformations of NleG2-3[90–191] domain.

Mapping of residues demonstrating significant chemical shift perturbation upon titration of UBE2D2 enzyme onto the NleG2-3[90–191] structure established that all these residues belong to the “core” RING/U-box portion of NleG2-3[90–191] (Figures 1 and 6B). Several residues that undergo significant chemical shift perturbations upon binding of UBE2D2cluster on the NleG2-3[90–191] surface in a shallow groove formed by NleG2-3[90–191] helix α3 and loops L2 and the N-terminus of L4 (Figures 6B and 6C, see residues 121–124, Asp145, Leu152 and Leu157). This NleG2-3[90–191] surface area corresponds to the common E2 binding site of eukaryotic RING/U-box domains, which was recently visualised in the structure of the CHIP E3 ligase U-box domain in complex with UBE2D2 [35]. The analogous surface region in the U-box motif of the *P. syringae* effector AvrPtoB, involving residues Phe479, Phe525 and Pro533 was shown to be part of the UBE2D2 E2 binding surface [19]. Superimposition of the AvrPtoB and NleG2-3[90–1919] structures (Figure 6D) showed that Phe479, Phe525 and Pro533 residues correspond to NleG2-3 conserved residues Ile121, Leu152, which demonstrate a significant line broadening upon binding of UBE2D2 protein and Pro160, respectively. These observations indicate that the E2 binding site might be conserved between eukaryotic, AvrPtoB and NleG U-boxes.

Single alanine mutation of Phe479, Phe525 or Pro533 residue in AvrPtoB has a dramatic effect on its interactions with UBE2D2 enzyme resulting in abolition of AvrPtoB autoubiquitination activity [19]. Accordingly we probed the effect of mutations of the corresponding conserved NleG residues on its *in vitro* ubiquitination activity. The NleG2-3 Ile121, Leu152 and Pro160 residues were individually substituted by alanine or lysine to generate a stronger effect. In addition we tested the effect of lysine substitution of NleG2-3 Leu123 and Asp145 residues. These two conserved and surface exposed residues also demonstrated significant chemical shift perturbation upon binding of UBE2D2 protein and are localized adjacent to the potential E2 binding area described for eukaryotic and AvrPtoB U-boxes (Figure 6A and B). All eight NleG2-3[90–191] point-mutation variants were tested for ubiquitination activity by our standard assay. According to the Western blotting analysis using anti-ubiquitin antibodies (Figure 6E), both alanine and lysine substitutions of NleG2-3 Ile121 and Leu152 residues led to significant decrease of ubiquitination activity resulting in diminishing in the formation of polyubiquitinated protein species. The effect was stronger in case of lysine substitutions of these NleG2-3 residues resulting in complete abrogation of formation of polyubiquitinated protein species at selected time point. In case of Pro160 mutations the P160K variant also demonstrated dramatic decrease in activity while P160A variant's activity was compatible with the wild type NleG2-3[90–191]. Taken together these results indicated that the NleG2-3 surface formed by these conserved residues corresponding to the common E2 binding site in eukaryotic RING/U-box and AvrPtoB E3 ligases might be directly involved in interactions with UBE2D2 enzyme. The lysine substitutions of NleG2-3 Leu123 and Asp145 did not significantly alter the *in vitro* ubiquitination activity compared to the wild type NleG proteins (Figure 6D). The change in chemical shifts of these residues upon titration of UBE2D2 protein described above may be indicative of a general conformational adjustment in the NleG2-3[90–191] molecule upon binding of E2 enzyme rather than of specific interactions of these NleG residues with the UBE2D2 protein.

**Figure 7:**
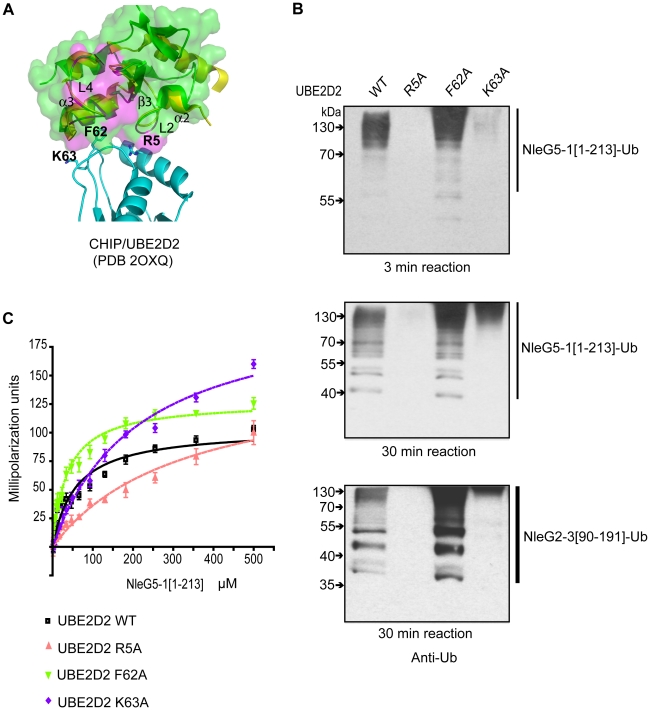
Characterisation of UBE2D2 surface interacting with NleG2-3 and NleG5-1. (A) Alignment of the E3 ligase domains of reduced NleG2-3[90–191] (green) with the CHIP U-box/UBE2D2 complex (PDB 2OXQ) (yellow for the CHIP U-box, cyan for the E2 enzyme), as shown using ribbon diagrams. The transparent surface of reduced NleG2-3[90–191] is also shown. As with Figure 6, residues in NleG2-3[90–191] whose NH resonances were severely line-broadened in ^1^N-^1^H HSQC spectrum upon addition of UBE2D2 are coloured purple. Selected residues in UBE2D2 are shown by a stick representation and labelled, and selected secondary structure elements on the NleG2-3[90–191] ribbon are also labelled. (B) Immunoblot analysis using anti-ubiquitin antibodies of reactions performed in the presence of ATP, ubiquitin, E1, NleG5-1[1–213] (top and middle panels) or NleG2-3[90–191] (lower panel) and UBE2D2 wild type (WT) or its R5A, F62A and K63A variants. Samples were incubated at 30°C for the indicated time. (**C**) Determination of dissociation constants of NleG5-1[1–213] interactions with UBE2D2 wild type (WT) or its R5A, F62A and K63A variants. The change in fluorescence polarisation of fluorescein-labelled UBE2D2 and its variants is plotted as a function of NleG5-1[1–213] concentration with error bars indicating standard deviation.

From the UBE2D2 perspective the interface involved in interaction with eukaryotic E3 ubiquitin ligases usually involves the N-terminal helix and loops 4 and 7 of this E2 enzyme. However, UBE2D2 specific contacts vary between different RING E3 ligases [36]. The individual mutations of Arg5, Phe62 and Lys63 in UBE2D2 residues making contact with the individual eukaryotic RING E3 ligase, had a dramatic effect on UBE2D2-E3 interactions, resulting in a decrease or complete loss of corresponding E3 ligase activity [35], [37]. To test if these mutations also affect NleG-UBE2D2 interactions, we examined the effect of Ala substitutions of Arg5, Phe62 and Lys63 in the UBE2D2 enzyme on the autoubiquitination activity of NleG2-3[90–191] and NleG5-1[1–213] proteins. Mutation of residues Arg5 and Lys63 to alanine significantly decreased the autoubiquitination activity of both NleG2-3[90–191] and NleG5-1[1–213] proteins (Figure 7B). The effect was more severe in the case of the R5A mutation, which almost completely abrogated the formation of polyubiquitinated species by both NleG proteins. The F62A substitution on the other hand had no noticeable effect on the activity of NleG2-3[90–191] and NleG5-1[1–213] proteins. In fact, this UBE2D2 mutant appeared to have higher activity than wild type UBE2D2 (Figure 7B).

To quantify the NleG5-1[1–213] interactions with UBE2D2 variants, we labelled the three UBE2D2 mutant proteins described above with fluorescein-5-maleimide and monitored the change in fluorescence polarization upon titration of NleG5-1[1–213] (Figure 7C). K_d_ values for NleG5-1[1–213] interactions with UBE2D2 R5A, F62A and K63A mutants were 326±35, 36±2 and 213±9 µM, respectively. Compared to the K_d_ for NleG5-1[1–213] interaction with wild type UBE2D2, presented above (56±2 µM) the affinity of R5A and K63A mutants were approximately 5 and 3 times lower, respectively, while the affinity of the F62A mutant was slightly higher. These affinity values correlate well with the autoubiquitination assay results. Taken together, these data suggest that the mutation of UBE2D2 Phe62 to Ala does not significantly affect the UBE2D2-NleG5-1 interactions. On the other hand, the R5A and K63A mutations in UBE2D2 disrupted the interaction with NleG2-3[90–191] and NleG5-1[1–213] proteins resulting in a concomitant decrease in autoubiquitination activity *in vitro*.

## Discussion

We have elucidated the structure and function for the first member of a large family of type 3 effectors collectively called NleG. The NleG family accounts for over 20 confirmed and potential effectors found primarily in pathogenic *E. coli* with fourteen members in the O157:H7 strain alone.

Our analysis demonstrated that all NleG effectors feature a conserved ∼100-residue region at the C-terminus which can be isolated as a soluble domain. The solution structure of this region from an NleG family member, NleG2-3 (NleG2-3[90–191]), revealed the presence of a motif structurally similar to the RING-finger and U-box domains characteristic of E3 ubiquitin ligases. Subsequent biochemical assays demonstrated that the full length and C-terminal portion of the NleG2-3 effectors are able to carry out autoubiquitination in the presence of human E1 and UBE2D2 E2 enzymes *in vitro*. Two other NleG effectors, namely NleG5-1 and NleG9, and their corresponding C-terminal domains also demonstrated strong autoubiquitination activity. Thus, the NleG family representatives share a common function reminiscent of eukaryotic RING-like E3 ubiquitin ligases.

NMR chemical shift perturbation experiments confirmed that the RING finger motif in NleG2-3[90–191] plays the primary role in interactions with the human E2 (UBE2D2) enzyme. Moreover several conserved NleG2-3[90–191] residues demonstrating significant chemical shift upon binding of UBE2D2 form a surface patch corresponding to a common E2 binding site of eukaryotic RING-like E3 ligases and that of the U-box domain of *P. syringae* effector AvrPtoB. Mutations in three conserved NleG residues that are part of this surface area result in dramatic decrease of NleG2-3[90–191] and NleG5-1[1–213] ubiquitination activity. On the E2 side of the E2/NleG interface, the R5A and K63A mutations of UBE2D2 had a deleterious effect on its ability to support the autoubiquitination activity of several RING/U-box proteins; analogous mutations had a similar effect on the activity of both NleG2-3 and NleG5-1. Overall the UBE2D2 interactions with NleG effectors appear to follow the general architecture established for E2/eukaryotic RING finger complexes.

Similar to other E3 ubiquitin ligases, the NleG2-3 effector demonstrated selectivity in recognition of E2 enzymes. Apart from UBE2D2 and its close homologues, only UBE2E2 and UBE2E3 were able to support the autoubiquitination activity of this protein out of 30 human E2 orthologs that were tested. Notably, the NleG5-1 effector, which shares only 32% sequence identity with NleG2-3, demonstrated a similar E2 specificity profile, indicating that preference in E2 enzymes may be conserved even among distant NleG homologues.

The significant structural similarity between NleG2-3[90–191] and the C-terminal domain (CTD) of the *P. syringae* AvrPtoB effector (PDB code 2FD4) is of particular interest. The CTD of AvrPtoB spans residues 436 to 553 (C-terminus) and, until this work, it represented the only structurally characterised bacterial effector domain featuring a U-box motif. The “core” U-box motifs in the AvrPtoB CTD and NleG2-3[90–191] superimpose with an r.m.s. deviation of 1.59 Å over 41 Cα atoms, while sharing only 18% sequence identity. Along with eukaryotic U-box domains, both AvrPtoB CTD and NleG2-3[90–191] structures lack the metal binding sites required for structural integrity of canonical RING domains. Both the NleG2-3[90–191] and AvrPtoB CTD domains express strong specificity toward the UBE2D2 family of E2 enzymes and demonstrate similarly located E2 binding sites as mentioned above. However the alanine substitutions of residues in AvrPtoB E2 binding sites appear to have a significantly stronger effect than similar mutations of equivalent residues in NleG2-3 and NleG5-1. These observations suggest that there may be subtle differences in the interactions of these proteins with the same E2 enzyme. Functional and structural similarities between AvrPtoB and NleG U-box domains raise a question of a potential evolutionary relationship between these bacterial effectors.

The RING-like E3 ligases promote the transfer of ubiquitin from E2 enzymes onto specific cellular targets. The recognition of specific target protein(s) is an essential part of this process and is usually performed by structural elements within the E3 ligase distinct from its RING finger or U-box. In the case of the AvrPtoB effector the recognition of its target, namely tomato Fen kinase was localised to the N-terminal domain (amino acids 1 to 387) of this protein [20]. The sequence analysis of full length NleG proteins and structural analysis of the NleG2-3[90–191] fragment presented in this work did not reveal any obvious candidates for a substrate protein binding motif. Further structural characterisation of full-length NleG family representatives that would include the variable N-terminal portion lacking in the current structure is required to gain insights into the potential location of substrate binding sites for this novel family of E3 ubiquitin ligases. The lack of a potential substrate binding motif in NleG effectors also opens an intriguing possibility that NleG effectors function may be primarily associated with binding to the specific host E2 enzyme rather than to the transfer of ubiquitin onto the substrate protein. Human zinc finger protein A20 (also known as TNFAIP3), which negatively regulates inflammatory signalling pathways, has been recently described to inhibit several E3 ligase activities by antagonizing interactions with the UBE2N (Ubc13) and UBE2D3 (UbcH5c) E2 enzymes [38]. A similar mechanism for the NleG effectors has yet to be demonstrated experimentally.

Structural and biochemical characterisation of NleG type 3 effectors as E3 ubiquitin ligases opens a new chapter in identification of their specific role in the host cell. The few effectors previously characterised with this activity are primarily involved in suppression of the host immune response by targeting immune-related host proteins to proteasome degradation. In *Shigella*, two diverse members of the IpaH effectors possessing E3 ubiquitin ligase activity were found to target different host proteins for ubiquitination [23]. The host targets of NleG effectors remain unknown. Identification of these targets represents the next challenge in unveiling the function of this important family of bacterial pathogenic factors.

## Materials and Methods

### DNA manipulation

His_6_-tagged constructs of all NleGs used in this study were amplified from *E. coli* H157:O7 strain Sakai genomic DNA and cloned into the T7 expression vector downstream of a DNA fragment encoding an N-terminal His_6_ tag followed by a TEV protease recognition and cleavage site.

Site-directed mutagenesis of NleG2-3, NleG5-1 and UBE2D2 was performed using the QuikChange site-directed mutagenesis kit (Stratagene) according to the manufacturer's protocol and verified by sequencing.

### Protein purification

His_6_-tagged NleGs and His_6_-tagged E2s were expressed and purified as previously described [39]. Briefly, the expression plasmid for each polypeptide was transformed into *Escherichia coli* BL21-Gold (DE3) (Stratagene), which harbours an extra plasmid (pMgk) encoding three rare tRNAs (AGG and AGA for arginine, and ATA for isoleucine). These *E. coli* cells were then cultured in 1 litre of Studier medium [40] supplemented with appropriate antibiotic (ampicillin (100 µg/ml), kanamycin (50 µg/ml) or chloramphenicol (25 µg/ml)), and incubated at 37°C for 4 hours, when the culture was allowed to grow overnight at 20°C. Cells were harvested by centrifugation, disrupted by sonication, and the insoluble material was removed by centrifugation. His_6_-tagged proteins were purified using nickel-nitrilotriacetic acid (Ni-NTA) affinity chromatography, dialyzed and stored in a buffer containing 10 mM HEPES, pH 7.5, 300 mM NaCl and 0.5 mM tris-(2-carboxyethyl) phophine (TCEP).

For NMR studies the His_6_-tagged NleG2-3[90–191] protein fragment was expressed in *E. coli* strain BL21-CodonPlus(DE3)-RIL (Stratagene). Cells were grown in 0.5 L of 2 X M9 minimal medium containing ^15^NH_4_Cl and ^13^C-glucose as the sole nitrogen and carbon source and supplemented with ZnSO_4_, thiamine, and biotin. The cells were grown at 37°C to an OD_600_ of 1.0 and protein expression was induced with 1 mM isopropyl β-D-thiogalactoside. The temperature was reduced to 15°C, and the cells were allowed to grow overnight before harvesting. Frozen cell pellets were thawed in 500 mM NaCl, 20 mM Tris, 5 mM imidazole (pH 8.0) and lysed by sonication. The proteins were extracted from the lysates by batch nickel affinity chromatography (Qiagen). The nickel affinity beads were washed three times with five column volumes of 500 mM NaCl, 20 mM Tris (pH 8.0), 30 mM imidazole, and the protein was eluted with five column volumes of 500 mM imidazole in this same buffer. The hexa-histidine tag was cleaved with TEV protease and the mixture passed through a nickel affinity column. The purified protein was concentrated, and buffer was exchanged by ultrafiltration and dilution/reconcentration into the NMR buffer containing 10 mM Tris (pH 7.0), 300 mM NaCl, 10 mM DTT, 1 mM benzamidine, 0.01% NaN_3_, 1 x inhibitor cocktail (Roche Applied Science), 95% H_2_O/5% D_2_O.

### NMR spectroscopy

The NMR experiments were carried out at 25°C on either Bruker Avance 600 or 800 MHz spectrometers equipped with cryogenic probes. All 3D spectra employed a non-uniform sampling scheme in the indirect dimensions and were reconstructed by multi-dimensional decomposition software MDD [41], [42], interfaced with NMRPipe [43]. The backbone assignments were obtained using HNCO, CBCA(CO)NH, HBHA(CO)NH, HNCA, and ^15^N-edited NOESY-HSQC spectra. Assignments were made initially with the automated program FAWN (Lemak and Arrowsmith, manuscript in preparation) and followed by manual analysis with SPARKY (http://cgl.ucsf.edu/home/sparky). Aliphatic side chain assignments relied on (H)CCH-TOCSY and H(C)CH-TOCOSY spectra [44], [45]. Aromatic ring resonances were assigned using 3D ^13^C-edited NOESY spectra. The tautomeric states of the histidines were determined by 2D long-range ^15^N-^1^H HSQC spectrum. Stereospecific valine and leucine methyl assignments were obtained as described [46] on the basis of the ^13^C-^13^C one-bond couplings in a high resolution 2D ^1^H-^13^C HSQC spectrum of 7%- ^13^C, 100%- ^15^N NleG2-3[90–191].

### Residual dipolar couplings

Three sets of residual dipolar couplings, namely *^1^D_NH_, ^1^D_CaCo_* and *^1^D_NCo_*, were measured on Bruker Avance 600 MHz spectrometer from interleaved HNCO-based in-phase/anti-phase (IP/AP) experiments on uniformly ^13^C, ^15^N-labelled NleG2-3[90–191] dispersed in buffer with and without alignment induced from 10 mg/mL Pf1 phages (D2O-splitting = 10 Hz) (ASLA, Riga, Latvia). The CαCo-coupled experiment was additionally acquired with BEST technology [47]. The NH-, CαCo- and NCo-coupled spectra were acquired with 320×32, 40×280 and 40×280 real + imaginary points, respectively, in the N and C indirect dimensions and all used the NUS scheme (30% reduction), followed by MDD reconstruction. The *^1^J* (isotropic sample) and (*^1^J+^1^D*) (anisotropic sample) couplings were measured from the separation between the two peaks corresponding to the *α* and *β* coherences and isolated in individual sub-spectra after linear combination of the IP and AP experiments using NMRPipe scripts. Peak positions and separation were evaluated in Sparky and validation was performed with Pales [48].

### Structure calculation

Distance restraints for structure calculations were derived from cross-peaks in ^15^N-edited NOESY-HSQC (τ_m_ = 100 ms) and ^13^C-edited aliphatic and aromatic NOESY-HSQC in H_2_O (τ_m_ = 100 ms), respectively. NOE peaks were picked and integrated with the program SPARKY. Automated NOE assignment and structure calculations were performed using the noeassign module implemented in the program CYANA, version 2.1 [49]. A total of 164 phi and psi torsion angle restraints for reduced NleG2-3[90–191] and 160 phi and psi torsion angle restraints for “oxidised” NleG2-3[90–191] were derived from the program TALOS [50]. Hydrogen bond restraints were applied only for residues that were clearly in the secondary structure regions as judged by NOE patterns and chemical shifts and supported by TALOS. In addition, disulfide bond restraints between Cys141 and Cys177 were imposed in the calculation of the “oxidised” conformation of NleG2-3[90–191]. A total of 94.2% of NOESY peaks were assigned for both the “reduced” and “oxidised” NleG2-3[90–191], respectively, in cycle 7. The quality of the noeassign/CYANA calculation was assessed by NMR structure quality assessment scores (NMR RPF scores) [51]. The best 20 of 100 CYANA structures from the final cycle were subjected to restrained molecular dynamics simulation in explicit water by the program CNS, which was modified to incorporate residual dipolar couplings [52], [53]. The final structures were inspected by PROCHECK [54] and MolProbity [55] using the NESG validation software package PSVS [56]. The validation reports are accessible at http://www.nesg.org. NleG2-3[90–191] is target ET109A of the Northeast Structural Genomics Consortium and the Midwest Center for Structural Genomics. Structures were visualised using the program MOLMOL [57] and Pymol (http://pymol.sourceforge.net, Delano Scientific).

### CS-Rosetta calculation

For reduced NleG2-3[90–191] model prediction, CS-Rosetta calculations [58] were performed in two steps. First, 10,000 CS-Rosetta models were generated. To evaluate the generated models we used both Rosetta energy and a score that measures compatibility of a model to unassigned NOESY peak lists. Then the model with the best-combined score was used as a starting structure in the second step to generate 2000 Rosetta models. The best cluster consisting of six models obtained on the second step was selected as a prediction. For oxidised NleG2-3[90–191] model prediction we generated 2000 model with CS-Rosetta using the reduced NleG2-3[90–191] model as a starting structure and applying disulfide bond restraints between residues Cys141 and Cys177.

### Titration studies of UBE2D2 and NleG2-3

Titration was performed by adding an aliquot of dilute unlabelled UBE2D2 in the NMR buffer to a 0.5 mL NMR sample of ^15^N-labelled NleG2-3[90–191], the diluted NMR sample was allowed to stand for at least an hour and then concentrated back to 0.5 mL. The ^15^N-HSQC spectra were recorded after each addition. Only two additions were made until an ∼1∶1 ratio of UBE2D2 and NleG2-3[90–191] was formed.

### In vitro assays

Ubiquitination reactions were performed in a 20-µl reaction mixture containing buffer (50 mM Tris·HCl (pH 7.5), 100 mM NaCl, 10 mM ATP, 10 mM MgCl_2_, 0.5 mM DTT), 4 µg of human ubiquitin (wild type and K″O″, Boston Biochem), 0.13 µg of E1, 2 µg of E2 and 2 µg of His_6_-tagged NleGs proteins. Reactions were incubated at 30°C for the indicated period of time and stopped by the addition of an equal volume of 2X Laemmli sample buffer (0.125 M Tris-HCl, pH 6.8, 20% glycerol, 4% SDS, 0.004% bromophenol blue, 100 mM DTT). Reaction mixtures were separated by SDS-PAGE, transferred onto a nitrocellulose membrane and probed with specific antibodies.

The collection of human E2 expression constructs was received as a gift from the S. Dhe-Paganon laboratory at the Structural Genomics Consortium (http://www.sgc.utoronto.ca/sgc-webpages/sgc-toronto.php). The constructs provided a T7 promoter-driven expression of the N-terminal poly-histidine fusion for each human E2 enzyme. The constructs were then transformed into *E. coli* Bl21-Gold (DE3) (Stratagene), expressed, and purified using Ni-NTA affinity chromatography as described above.

### Fluorescence polarization assay

The UBE2D2 protein and its R5A, F62A and K63A variants were each incubated with fluorescein-5-maleimide (Molecular Probes) at a 1:25 molar ratio of E2 to fluorescein-5-maleimide at 4°C for 12 h. Free fluorescent dye was removed by gel-filtration chromatography followed by dialysis. The completeness of labelling was confirmed by mass spectroscopy. A starting concentration of the labelled E2 variants of 1 nM was selected according to the extent of conjugated fluorophore. Binding assays with NleG5-1 variants were performed in the buffer containing 25 mM HEPES, pH 7.5, 0.15 M NaCl, and 0.5 mM TCEP. Fluorescence anisotropy was measured at 25°C using a “Synergy 2” fluorescence polarization instrument (Biotek) with the excitation wavelength set at 485 nm and emission wavelength set at 528 nm. Increasing amounts of NleGs were added to aliquots of labelled E2 protein. Data from four measurements were averaged and fitted to a single-site binding model using nonlinear regression with GraphPad Prism 4 (version 4.00 for Windows, GraphPad Software, San Diego California USA, http://www.graphpad.com).

### Accession codes

The structures of NleG2-3[90–191] in both reduced and oxidised conformations has been deposited at the Protein Data Bank with accession codes 2KKX and 2KKY, respectively.

## Supporting Information

Table S1 Official nomenclature and most common synonym used for E2 ubiquitin conjugating enzymes. The E2 nomenclature is in accordance with that used by the Human Genome Organization (http://www.genenames.org/genefamily/ube2.php).
(0.06 MB DOC)

Figure S1 ^1^H-^15^N HSQC spectra of NleG2-3[90-191] and mutants (A) NleG2-3[90–191] (blue) and NleG2-3[90–191] C141A (red) (B) NleG2-3[90-191] (blue) and NleG2-3[90–191] C177A (magenta).
(0.99 MB TIF)

Figure S2 Plots of experimental vs. calculated RDCs for NleG2-3[90-191] after including RDC restraints in refinement. The experimental *^1^D_NH_*, *^1^D_CaCo_* and *^1^D_NCo_* values, shown on the ordinate were measured at 25°C using 10 mg/mL Pf1 phages at pH 7.0. The calculated RDC values, shown on the abscissa, were determined using the coordinates of lowest energy structure of reduced or oxidized NleG2-3[90–191].
(0.72 MB TIF)

Figure S3 Characterisation of zinc binding interactions with NleG2-3[90–191]. (A) Overlay of reduced NleG2-3[90–191](green) with RING finger 38 protein (PDB 1X4J)(red). Residues of RING finger 38 involved in binding the 2 Zn ions (shown as grey spheres) are shown in a stick representation and labelled, as are the corresponding residues in the NleG C-terminal domain. (B) Overlay of the ^1^H-^15^N HSQC spectra of NleG2-3[90–191] with (red) and without (blue) adding Zn^+2^ to the sample.
(2.60 MB TIF)

## References

[1] Galan JE (2009). Common themes in the design and function of bacterial effectors.. Cell Host Microbe.

[2] Parsot C (2009). *Shigella* type III secretion effectors: how, where, when, for what purposes?. Curr Opin Microbiol.

[3] Bhavsar AP, Guttman JA, Finlay BB (2007). Manipulation of host-cell pathways by bacterial pathogens.. Nature.

[4] Sal-Man N, Biemans-Oldehinkel E, Finlay BB (2009). Structural microengineers: pathogenic *Escherichia coli* redesigns the actin cytoskeleton in host cells.. Structure.

[5] Mounier J, Popoff MR, Enninga J, Frame MC, Sansonetti PJ (2009). The IpaC carboxyterminal effector domain mediates Src-dependent actin polymerization during *Shigella* invasion of epithelial cells.. PLoS Pathog.

[6] Steele-Mortimer O (2008). The *Salmonella*-containing vacuole: moving with the times.. Curr Opin Microbiol.

[7] Alto NM, Weflen AW, Rardin MJ, Yarar D, Lazar CS (2007). The type III effector EspF coordinates membrane trafficking by the spatiotemporal activation of two eukaryotic signaling pathways.. J Cell Biol.

[8] Brodsky IE, Medzhitov R (2009). Targeting of immune signalling networks by bacterial pathogens.. Nat Cell Biol.

[9] Navarro L, Alto NM, Dixon JE (2005). Functions of the *Yersinia* effector proteins in inhibiting host immune responses.. Curr Opin Microbiol.

[10] Rytkonen A, Holden DW (2007). Bacterial interference of ubiquitination and deubiquitination.. Cell Host Microbe.

[11] Angot A, Vergunst A, Genin S, Peeters N (2007). Exploitation of eukaryotic ubiquitin signaling pathways by effectors translocated by bacterial type III and type IV secretion systems.. PLoS Pathog.

[12] Spallek T, Robatzek S, Gohre V (2009). How microbes utilize host ubiquitination.. Cell Microbiol.

[13] Huibregtse JM, Scheffner M, Beaudenon S, Howley PM (1995). A family of proteins structurally and functionally related to the E6-AP ubiquitin-protein ligase.. Proc Natl Acad Sci U S A.

[14] Schwarz SE, Rosa JL, Scheffner M (1998). Characterization of human HECT domain family members and their interaction with UbcH5 and UbcH7.. J Biol Chem.

[15] Scheffner M, Nuber U, Huibregtse JM (1995). Protein ubiquitination involving an E1-E2-E3 enzyme ubiquitin thioester cascade.. Nature.

[16] Jackson PK, Eldridge AG, Freed E, Furstenthal L, Hsu JY (2000). The lore of the RINGs: substrate recognition and catalysis by ubiquitin ligases.. Trends Cell Biol.

[17] Cyr DM, Hohfeld J, Patterson C (2002). Protein quality control: U-box-containing E3 ubiquitin ligases join the fold.. Trends Biochem Sci.

[18] Abramovitch RB, Martin GB (2005). AvrPtoB: a bacterial type III effector that both elicits and suppresses programmed cell death associated with plant immunity.. FEMS Microbiol Lett.

[19] Janjusevic R, Abramovitch RB, Martin GB, Stebbins CE (2006). A bacterial inhibitor of host programmed cell death defenses is an E3 ubiquitin ligase.. Science.

[20] Rosebrock TR, Zeng L, Brady JJ, Abramovitch RB, Xiao F (2007). A bacterial E3 ubiquitin ligase targets a host protein kinase to disrupt plant immunity.. Nature.

[21] Angot A, Peeters N, Lechner E, Vailleau F, Baud C (2006). *Ralstonia solanacearum* requires F-box-like domain-containing type III effectors to promote disease on several host plants.. Proc Natl Acad Sci U S A.

[22] Diao J, Zhang Y, Huibregtse JM, Zhou D, Chen J (2008). Crystal structure of SopA, a *Salmonella* effector protein mimicking a eukaryotic ubiquitin ligase.. Nat Struct Mol Biol.

[23] Rohde JR, Breitkreutz A, Chenal A, Sansonetti PJ, Parsot C (2007). Type III secretion effectors of the IpaH family are E3 ubiquitin ligases.. Cell Host Microbe.

[24] Haraga A, Miller SI (2006). A *Salmonella* type III secretion effector interacts with the mammalian serine/threonine protein kinase PKN1.. Cell Microbiol.

[25] Zhu Y, Li H, Hu L, Wang J, Zhou Y (2008). Structure of a *Shigella* effector reveals a new class of ubiquitin ligases.. Nat Struct Mol Biol.

[26] Singer AU, Rohde JR, Lam R, Skarina T, Kagan O (2008). Structure of the *Shigella* T3SS effector IpaH defines a new class of E3 ubiquitin ligases.. Nat Struct Mol Biol.

[27] Quezada CM, Hicks SW, Galan JE, Stebbins CE (2009). A family of *Salmonella* virulence factors functions as a distinct class of autoregulated E3 ubiquitin ligases.. Proc Natl Acad Sci U S A.

[28] Kaper JB, McDaniel TK, Jarvis KG, Gomez-Duarte O (1997). Genetics of virulence of enteropathogenic *E. coli*.. Adv Exp Med Biol.

[29] Dean P, Maresca M, Kenny B (2005). EPEC's weapons of mass subversion.. Curr Opin Microbiol.

[30] Tobe T, Beatson SA, Taniguchi H, Abe H, Bailey CM (2006). An extensive repertoire of type III secretion effectors in *Escherichia coli* O157 and the role of lambdoid phages in their dissemination.. Proc Natl Acad Sci U S A.

[31] Li M, Rosenshine I, Yu HB, Nadler C, Mills E (2006). Identification and characterization of NleI, a new non-LEE-encoded effector of enteropathogenic *Escherichia coli* (EPEC).. Microbes Infect.

[32] Holm L, Kaariainen S, Rosenstrom P, Schenkel A (2008). Searching protein structure databases with DaliLite v.3.. Bioinformatics.

[33] Capili AD, Edghill EL, Wu K, Borden KL (2004). Structure of the C-terminal RING finger from a RING-IBR-RING/TRIAD motif reveals a novel zinc-binding domain distinct from a RING.. J Mol Biol.

[34] Lorick KL, Jensen JP, Fang S, Ong AM, Hatakeyama S (1999). RING fingers mediate ubiquitin-conjugating enzyme (E2)-dependent ubiquitination.. Proc Natl Acad Sci U S A.

[35] Xu Z, Kohli E, Devlin KI, Bold M, Nix JC (2008). Interactions between the quality control ubiquitin ligase CHIP and ubiquitin conjugating enzymes.. BMC Struct Biol.

[36] Deshaies RJ, Joazeiro CA (2009). RING domain E3 ubiquitin ligases.. Annu Rev Biochem.

[37] Winkler GS, Albert TK, Dominguez C, Legtenberg YI, Boelens R (2004). An altered-specificity ubiquitin-conjugating enzyme/ubiquitin-protein ligase pair.. J Mol Biol.

[38] Shembade N, Ma A, Harhaj EW Inhibition of NF-κB signaling by A20 through disruption of ubiquitin enzyme complexes.. Science.

[39] Zhang RG, Skarina T, Katz JE, Beasley S, Khachatryan A (2001). Structure of *Thermotoga maritima* stationary phase survival protein SurE: a novel acid phosphatase.. Structure.

[40] Studier FW (2005). Protein production by auto-induction in high density shaking cultures.. Protein Expr Purif.

[41] Gutmanas A, Jarvoll P, Orekhov VY, Billeter M (2002). Three-way decomposition of a complete 3D ^15^N-NOESY-HSQC.. J Biomol NMR.

[42] Luan T, Jaravine V, Yee A, Arrowsmith CH, Orekhov VY (2005). Optimization of resolution and sensitivity of 4D NOESY using multi-dimensional decomposition.. J Biomol NMR.

[43] Delaglio F, Grzesiek S, Vuister GW, Zhu G, Pfeifer J (1995). NMRPipe: a multidimensional spectral processing system based on UNIX pipes.. J Biomol NMR.

[44] Bax A, Vuister GW, Grzesiek S, Delaglio F, Wang AC (1994). Measurement of homo- and heteronuclear J couplings from quantitative J correlation.. Methods Enzymol.

[45] Kay LE (1997). NMR methods for the study of protein structure and dynamics.. Biochem Cell Biol.

[46] Neri D, Szyperski T, Otting G, Senn H, Wüthrich K (1989). Stereospecific nuclear magnetic resonance assignments of the methyl groups of valine and leucine in the DNA-binding domain of the 434 repressor by biosynthetically directed fractional ^13^C labeling.. Biochemistry.

[47] Lescop E, Schanda P, Brutscher B (2007). A set of BEST triple-resonance experiments for time-optimized protein resonance assignment.. J Magn Reson.

[48] Cornilescu G, Marquardt JL,  O M, Bax A (1998). Validation of protein structure from anisotropic carbonyl chemical shifts in a dilute liquid crystalline phase.. J Am Chem Soc.

[49] Guntert P (2004). Automated NMR structure calculation with CYANA.. Methods Mol Biol.

[50] Cornilescu G, Delaglio F, Bax A (1999). Protein backbone angle restraints from searching a database for chemical shift and sequence homology.. J Biomol NMR.

[51] Huang YJ, Powers R, Montelione GT (2005). Protein NMR recall, precision, and F-measure scores (RPF scores): structure quality assessment measures based on information retrieval statistics.. J Am Chem Soc.

[52] Brunger AT, Adams PD, Clore GM, DeLano WL, Gros P (1998). Crystallography & NMR system: A new software suite for macromolecular structure determination.. Acta Crystallogr D Biol Crystallogr.

[53] Linge JP, Williams MA, Spronk CA, Bonvin AM, Nilges M (2003). Refinement of protein structures in explicit solvent.. Proteins.

[54] Laskowski RA, Rullmannn JA, MacArthur MW, Kaptein R, Thornton JM (1996). AQUA and PROCHECK-NMR: programs for checking the quality of protein structures solved by NMR.. J Biomol NMR.

[55] Lovell SC, Davis IW, Arendall WB, de Bakker PI, Word JM (2003). Structure validation by Cα geometry: ϕ, ψ and Cβ deviation.. Proteins.

[56] Bhattacharya A, Tejero R, Montelione GT (2007). Evaluating protein structures determined by structural genomics consortia.. Proteins.

[57] Koradi R, Billeter M, Wuthrich K (1996). MOLMOL: a program for display and analysis of macromolecular structures.. J Mol Graph.

[58] Shen Y, Lange O, Delaglio F, Rossi P, Aramini JM (2008). Consistent blind protein structure generation from NMR chemical shift data.. Proc Natl Acad Sci U S A.

[59] Chenna R, Sugawara H, Koike T, Lopez R, Gibson TJ (2003). Multiple sequence alignment with the Clustal series of programs.. Nucleic Acids Res.

