# Supplementary material for: NleG Type 3 Effectors from Enterohaemorrhagic Escherichia coli Are U-Box E3 Ubiquitin Ligases
Source: PLoS Pathog. 2010 Jun 24;6(6):e1000960. doi: 10.1371/journal.ppat.1000960 (PMC2891834; doi:10.1371/journal.ppat.1000960)
Supplement: Table S1 — Official nomenclature and most common synonym used for E2 ubiquitin conjugating enzymes. The E2 nomenclature is in accordance with that used by the Human Genome Organization (http://www.genenames.org/genefamily/ube2.php). (0.06 MB DOC) [file ppat.1000960.s001.doc]

Supplementary Table 1**.** Official nomenclature and most common synonym used for E2 ubiquitin conjugating enzymes. The E2 nomenclature is in accordance with that used by the Human Genome Organization (http://www.genenames.org/genefamily/ube2.php).

| Human Genome Organization Nomenclature | Synonym |
| --- | --- |
| UBE2V2 | UEV2/MMS2 |
| UBE2D1 | UBC4/5/UBCH5A |
| UBE2D2 | UBC4/5/UBCH5B |
| UBE2D4 | HBUCE1 |
| UBE2D3 | UBC4/5 |
| UBE2W | FLJ11011 |
| UBE2B | UBC2/HHR6B/RAD6B/E217K |
| UBE2L6 | RIGB/UBCH8 |
| UBE2N | UBC13 |
| UBE2L3 | UBCH7 |
| UBE2G1 | UBC7/E217K |
| UBE2H | UBC8/E220K |
| UBE2M | UBC12 |
| UBE2F | NCE2 |
| UBE2E2 | UBCH8 |
| UBE2E3 | UBCH9/UBCM2 |
| UBE2S | E224K |
| UBE2U | MGC35130 |
| UBE2R1 | CDC34 |
| UBE2R2 | UBC3B/CDC34B |
| UBE2Z | HOYS7 |
| UBE2J2 | NCUBE2 |
| Probable ubiquitin-conjugating enzyme E2 FLJ25076 | LOC134111/FLJ25076 |
| AKTIP | FTS/FT1 |
| UBE2J1 | NCUBE1 |
| UBE2V1 | UEV1/CROC1 |
| UBE2Q2 | DKFZ/UBCI |
| UBE2Q1 | NICE5 |
|  | TSG101/VPS23/SG10 |
| UEVLD | UEV3 |
