# Supplementary figures and images for: NleG Type 3 Effectors from Enterohaemorrhagic Escherichia coli Are U-Box E3 Ubiquitin Ligases
Source: PLoS Pathog. 2010 Jun 24;6(6):e1000960. doi: 10.1371/journal.ppat.1000960 (PMC2891834; doi:10.1371/journal.ppat.1000960)

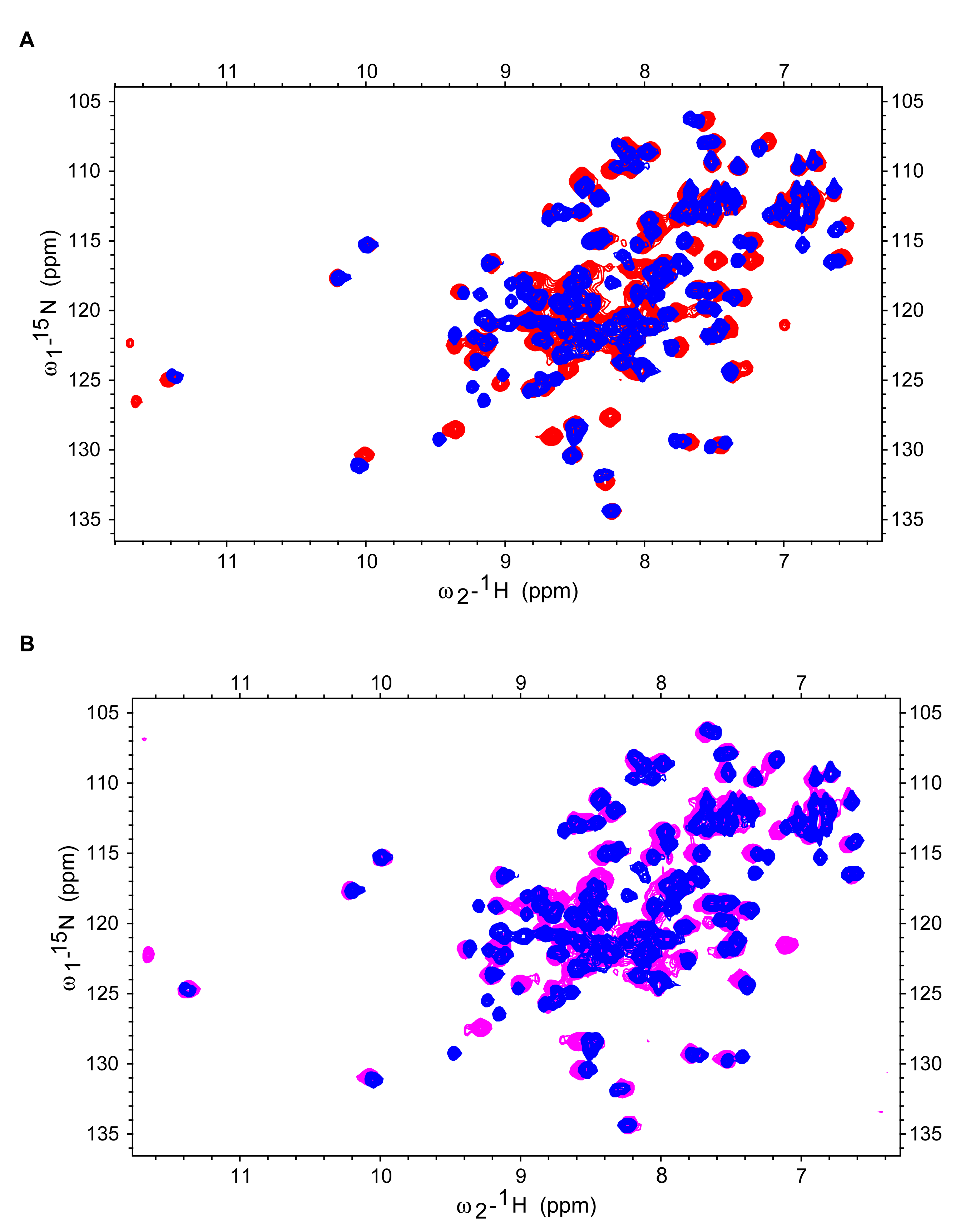

Supplement: Figure S1 — 1H-15N HSQC spectra of NleG2-3[90-191] and mutants (A) NleG2-3[90–191] (blue) and NleG2-3[90–191] C141A (red) (B) NleG2-3[90-191] (blue) and NleG2-3[90–191] C177A (magenta). (0.99 MB TIF) [file ppat.1000960.s002.tif]

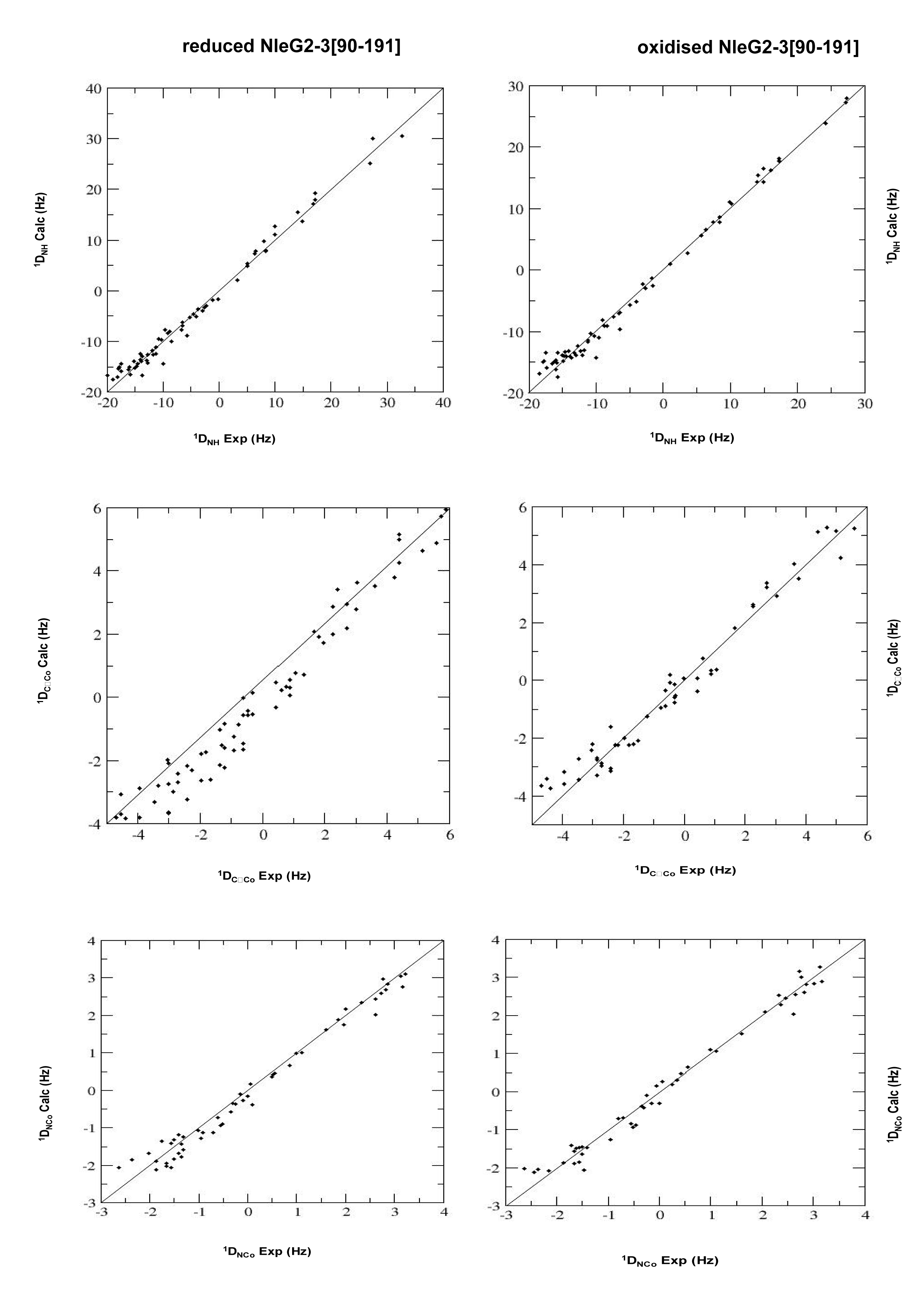

Supplement: Figure S2 — Plots of experimental vs. calculated RDCs for NleG2-3[90-191] after including RDC restraints in refinement. The experimental 1DNH, 1DCaCo and 1DNCo values, shown on the ordinate were measured at 25°C using 10 mg/mL Pf1 phages at pH 7.0. The calculated RDC values, shown on the abscissa, were determined using the coordinates of lowest energy structure of reduced or oxidized NleG2-3[90–191]. (0.72 MB TIF) [file ppat.1000960.s003.tif]

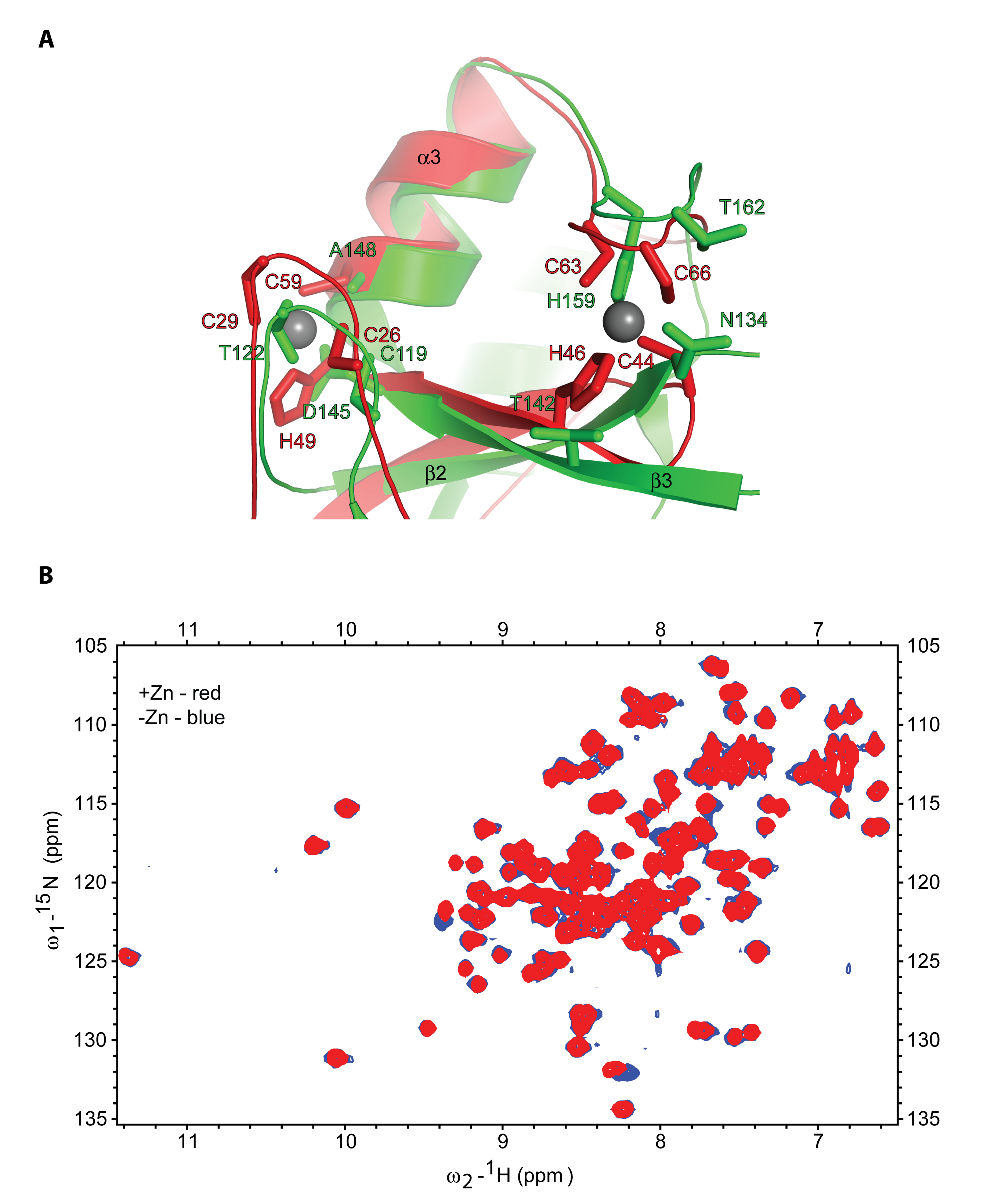

Supplement: Figure S3 — Characterisation of zinc binding interactions with NleG2-3[90–191]. (A) Overlay of reduced NleG2-3[90–191](green) with RING finger 38 protein (PDB 1X4J)(red). Residues of RING finger 38 involved in binding the 2 Zn ions (shown as grey spheres) are shown in a stick representation and labelled, as are the corresponding residues in the NleG C-terminal domain. (B) Overlay of the 1H-15N HSQC spectra of NleG2-3[90–191] with (red) and without (blue) adding Zn+2 to the sample. (2.60 MB TIF) [file ppat.1000960.s004.tif]
